# Newest Methods and Approaches to Enhance the Performance of Optical Frequency-Domain Reflectometers

**DOI:** 10.3390/s24165432

**Published:** 2024-08-22

**Authors:** Ivan A. Lobach, Andrei A. Fotiadi, Vasily A. Yatseev, Yuri A. Konstantinov, Fedor L. Barkov, D. Claude, Dmitry A. Kambur, Maxim E. Belokrylov, Artem T. Turov, Dmitry A. Korobko

**Affiliations:** 1 Institute of Automation and Electrometry, Siberian Branch, Russian Academy of Sciences, 630090 Novosibirsk, Russia; lobach@iae.nsk.su; 2 Electromagnetism and Telecommunication Department, University of Mons, 7000 Mons, Belgium; 3 S.P. Kapitsa Research Institute of Technology, Ulyanovsk State University, 42 Leo Tolstoy Street, 432970 Ulyanovsk, Russia; korobkotam@rambler.ru; 4 Kotelnikov Institute of Radioengineering and Electronics of Russian Academy of Science, 125009 Moscow, Russia; yatseev@gmail.com; 5 Institute of Continuous Media Mechanics, Ural Branch, Russian Academy of Sciences, 1 Acad. Korolev Street, 614018 Perm, Russia; fbarkov@pstu.ru (F.L.B.); cdf750@yandex.ru (D.C.); kambur.dima@gmail.com (D.A.K.); belokrylovme@gmail.com (M.E.B.); artemtur442@gmail.com (A.T.T.); 6 Applied Mathematics Department, Perm National Research Polytechnic University, Komsomolsky Avenue 29, 614990 Perm, Russia; 7 Optical Reflectometry Metrology and Sensing Laboratory, LLC, Komsomolsky Avenue 69, 614039 Perm, Russia; 8 General Physics Department, Perm National Research Polytechnic University, Komsomolsky Avenue 29, 614990 Perm, Russia

**Keywords:** frequency domain, OFDR, fiber optic sensing, distributed fiber optic sensing, metrology, strain, temperature, backscattering, high resolution, demodulation, algorithms, cross-correlation, auxiliary interferometer, nonlinearity compensation, laser sweep

## Abstract

In this review, we summarize the latest advances in the design of optical frequency-domain reflectometers (OFDRs), digital signal processing, and sensors based on special optical fibers. We discuss state-of-the-art approaches to improving metrological characteristics, such as spatial resolution, SNR, dynamic range, and the accuracy of determining back reflection coefficients. We also analyze the latest achievements in the OFDR-based sensors: the accuracy of spatial localization of the impact, the error in detecting temperatures, deformation, and other quantities, and the features of separate measurement of various physical quantities. We also pay attention to the trend of mutual integration of frequency-domain optical reflectometry methods with time-domain optical reflectometry, which provides completely new sensing possibilities. We believe that this review may be useful to engineers and scientists focused on developing a lab setup, complete measurement instrument, or sensing system with specific requirements.

## 1. Introduction

**Figure 1:**
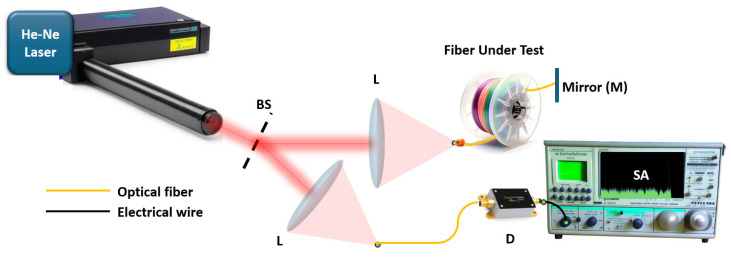
The setup proposed in [1].

In 1981, Eickhoff and Ulrich, two German researchers from the Technical University of Hamburg, demonstrated a new method of scattering and reflection registration along the length of an optical fiber. The method was based on fundamentally new approaches [1]. In contrast to the well-known optical time-domain reflectometry (OTDR) quite widely used at that time to assess the integrity of relatively long fragments of optical fibers, the authors used continuous radiation probing. Its optical frequency linearly changes over time. The setup (Figure 1) consisted of a single-frequency He-Ne laser, a beam splitter BS, a pair of lenses L, a detector D, a radio frequency spectrum analyzer SA, and a mirror at the end of the fiber line M. Coming into the splitter BS, each backscattered component corresponding to a specific location in the fiber interfered with the radiation of contrast reflection from the mirror M.

Due to a time delay between each such pair of waves corresponding to a specific coordinate along the fiber, radiation arrived at the splitter with a certain frequency difference, recorded in the form of interference beats via the detector D. Since the mirror was located at the far end of the line, the coordinate region was inverted relative to the beat-frequency region. However, it established a clear connection between the coordinate and the frequency obtained via the SA. This experiment demonstrated the principle of an OFDR for the first time. One can understand how OFDR works using simple analogies. For example, a siren from an emergency vehicle, which also has frequency tuning, but in the range of acoustic frequencies, at a far distance from the observer, creates the feeling of a full convoy of similar vehicles moving. This is explained by the fact that in a large city, a sound wave is reflected from various buildings and structures located at different distances from both the sound source and the observer. Sound intervals, that is, the ratios of mixed sound frequencies, can be determined via the human ear—especially those with an ear for music. In fact, the frequency Interval will determine the distance from the car to the observer. The role of “musical hearing” in Figure 1 is performed via an electronic spectrum analyzer. That is exactly why this method is called optical frequency-domain reflectometry—the frequency is determining the quantity in it. A high spatial resolution, independent from the pulsed nature of the system’s probing radiation, has become its main advantage. This made it possible to study not only fiber optic circuits but also integrated optical chips using OFDR. In addition, using both the amplitude and phase spectra, it is possible to interpret various effects on the fiber. So, another perspective area has been opened up for the technology: fiber sensors.

Of course, over the past 40 plus years, other distributed fiber optic sensors and metrology technologies have not stood still: low- and high-coherence OTDR-based sensors (COTDR and φ-OTDR) [2,3,4,5,6,7], distributed fiber optic sensors based on Brillouin scattering (BOTDA and BOTDR) [8,9,10,11,12,13], and other reflectometer and sensor types [14,15,16,17] have continued to develop. However, despite the competition between technologies, the OFDR method has retained its position due to its high resolution, as well as its integration into the field of acoustic frequency monitoring and other dynamically developing areas of science and technology [18,19,20]. Currently, the method is actively used in the studies of new products designs [21], new materials [22], structures [23], vehicle tests [24], and shape sensors [25]. The study of photonic integrated circuits’ internal structure via reflectometry is still possible using OFDR technology only. Despite the described advantages, the technology has some shortcomings, and developers face a number of crucial challenges. For example, in commercial samples of OFDRs, the option of thermal and deformation effects separation in fiber optic sensing is still a huge rarity. Even if it is present, the separation is carried out rather roughly. In addition, widespread integration into various fields of science and technology is limited due to the rather high cost of systems, caused by a need for a coherent tunable laser source (TLS) and some other components. Also, the problems of polarization fading suppression and the appearance of phantom bursts on OFDR traces have not been completely resolved. These undesirable phenomena lead to limitation of metrological, sensing, and other operational parameters of the system. Over all these years, researchers from all over the world have been gradually approaching solutions to these problems. The purpose of this review article is to show the progress of various research groups in improving the metrological and sensor performance of systems based on OFDRs, achieved using state-of-the-art software and hardware approaches over the past several years.

The reason why metrological parameters are explicitly separated from sensing ones is that for some researchers involved in the study of optical fibers samples and photonic chips, as well as their joints, the parameters of back reflection sensitivity and spatial sampling are very important for the measurement of back reflections. Of course, this spatial sampling will determine the spatial resolution of the OFDR when operating in sensing mode, but since these quantities are not the same, they must be defined separately. In general, the directions for OFDR characteristic improvements presented in this review are delineated based on applications (sections) and implementation methods (in some sections—subsections). So, the structure of this work is as follows. After the introduction (Section 1), basic theoretical information about the operation of the method (Section 2) is provided. The narration is told in laconic engineering language, which is suitable in order to understand the practical essence of the method. In Section 3, the basic equations and principles are derived by which the performance characteristics of a system based on frequency-domain optical reflectometry can be assessed. This section is considered to be quite important for people designing a real research stand or a commercial device based on OFDR. Section 4 is devoted to the main measures of the metrological properties of research systems based on the improvement of frequency-domain reflectometers. These primarily include the spatial resolution as well as the range and accuracy of back reflections recording. This part of this review is worth paying attention to for researchers involved in the creation and study of photonic integrated circuits, as well as their coupling with various optical fibers. Also, this section considers compensation for the nonlinearity of laser source tuning using various methods. Section 5 is devoted to the latest changes in the software and hardware of the systems, which have led to improved sensing parameters: sensitivity to temperatures and deformations, increased ranges of recorded values, data acquisition speed, and so on. The section begins with the basics of the method for extracting temperatures and strains from OFDR data. Section 6 continues the sensing theme to some extent, since it is devoted to the separate measurement of various physical quantities. In the most common case, these two quantities are temperature and strain. First of all, the patent, which represented pioneering research in its early years, is discussed. Next, the works that in recent years have made a great contribution to solving this problem using modern methods, including neural networks, are presented. Section 7 in some ways stands apart from the rest of this review because it does not indicate any specific application or set of specific parameters that are being improved. This part of this review will discuss the mutual integration of OTDR and OFDR technologies, which opens up new research opportunities. In Section 8, solutions to the problem of OFDR-based systems’ cost reduction are described. As mentioned above, the key and at the same time the most expensive part of the system is usually the TLS; therefore, in this section, special attention is paid to replacing this element with various devices operating on different principles. Section 9 is, in a sense, this work’s result; it summarizes all the material considered, compares technologies, and evaluates the immediate prospects for the development of the OFDR method.

## 2. Theory

**Figure 2:**
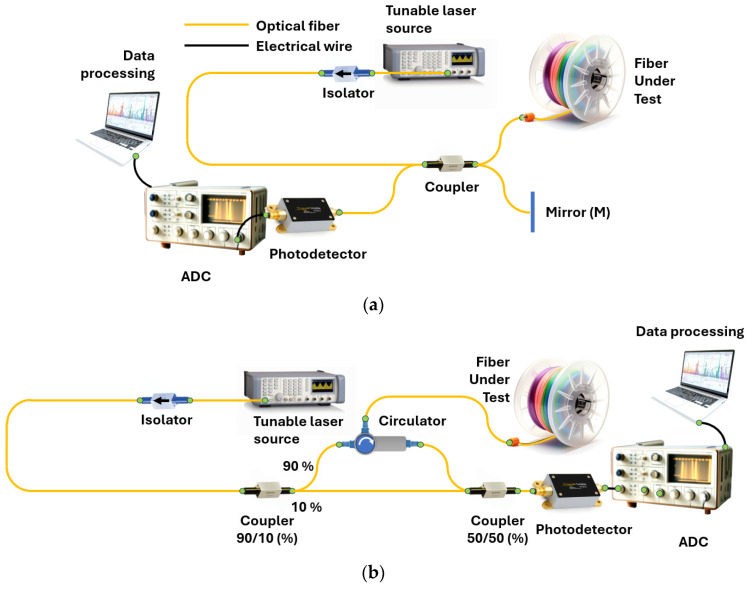
OFDR based on (**a**) Michelson and (**b**) Mach–Zehnder interferometer.

In most cases, the term OFDR refers to reflectometry based on a highly coherent frequency-tunable radiation source used as a probe radiation. In this case, to obtain information about the fiber path, it is inserted into one of the arms of a two-beam interferometer: Michelson (Figure 2a) or Mach–Zehnder (Figure 2b). To form a Michelson interferometer, it is sufficient to have one fiber coupler with a probe source and a photodetector on one side, and the FUT and mirror on the other. For a Mach–Zehnder interferometer, two fiber couplers are required—one for splitting the probe radiation into two paths, and the second for joining them. The result of the coupling is measured using a photodetector. The FUT is inserted into one of the interferometer arms utilizing a circulator (or also a fiber coupler). The valuable information in both cases is the interference product of two monochromatic signals that differ slightly in frequency and/or phase (due to frequency tuning and the time difference between a specific scattering center output and the reference signal). The main task of such a setup is to measure the spectral transmission function of the interferometer including the FUT.

Next, an expression for the signal reaching the photodetector can be derived. Without loss of generality, OFDR based on the Michelson interferometer will be considered. There is the interference of two waves in the photodetector: reflected from the mirror and backscattered from a certain point with the coordinate *z* of the FUT (Figure 2). Let the optical frequency of the laser radiation change in time linearly with the speed *γ*:

(1) $$\nu (t)=\nu_{0}+\gamma t,$$

where *ν*_0_ is the starting frequency of the laser tuning. Then, the electric field of the wave reflected from the mirror will be expressed as the relation

(2) $$E_{r}(t)=\sqrt{r}E_{0}k_{1}e^{j(2\pi \nu_{0}t+\pi \gamma t^{2})},$$

where *j* is the imaginary unit, *r* is the mirror reflectivity, *E*_0_ is the amplitude of the source wave field, and *k*_1_ is the coefficient determined via the coupler used. Similarly, the electric field of the wave backscattered from point *X* is expressed as the relation

(3) $$E_{S}(t)=\sqrt{R(\tau )}E_{0}k_{2}e^{j(2\pi \nu_{0}(t-\tau )+\pi \gamma {\left(t-\tau \right)}^{2})},$$

where *R*(*τ*) is the backscattering coefficient at a point with coordinate *z*, *k*_2_ is the coefficient determined via the coupler used, and $\tau =\frac{2z}{\upsilon }$ is the time delay between two waves determined by the difference in paths *z* between the two arms and the speed of light in the fiber *υ*.

The intensity of the electric field when coupling the two waves will be equal to

(4) $$I={\left|E_{r}+E_{S}\right|}^{2}=E_{r}^{2}+E_{S}^{2}+2E_{r}E_{S}\cos\varphi (t),$$

where *φ*(*t*) = *φ*_0_ + 2*πτγt* is the phase difference between the two waves; $\varphi_{0}=2\pi \tau (\nu_{0}-\frac{\gamma \tau }{2}).$

It can be seen that the final intensity consists of constant *I_DC_* and variable *I_AC_* components, which have the form

(5) $$I_{DC}=I_{0}\left\{rk_{1}^{2}+R(\tau )k_{2}^{2}\right\}$$

(6) $$I_{AC}=2\sqrt{rR(\tau )}k_{1}k_{2}I_{0}\cos(\varphi_{0}+2\pi \tau \gamma t)$$

For one pointwise reflector, the signal from the photodetectors will have a harmonic form with a modulation frequency of *ξ* = *γτ*. To determine the position of the reflector in the FUT, it is sufficient to apply the Fourier transform to the photodetector’s signal, which makes it possible to determine the modulation frequency *ξ* and then calculate *z* = *ξυ*/2*γ*. The value of the Fourier amplitude will be determined via the visibility of the interferometric pattern

(7) $$V=\frac{I_{\max}-I_{\min}}{I_{\max}+I_{\min}}=\frac{\sqrt{rR(\tau )}k_{1}k_{2}}{rk_{1}^{2}+R(\tau )k_{2}^{2}},$$

where *I*_max_ and *I*_min_ are the maximum and minimum values of the recorded intensity. It can be seen from the expression that the Fourier amplitude at the frequency *ξ* = *γτ* is determined by the reflection coefficient of the point reflector *R*(*τ*). However, it should also be noted that the amplitude depends on the interferometer parameters—the mirror reflection coefficient and the splitting coefficient of the coupler. Thus, to measure the absolute value of the reflector’s reflection coefficient, it is necessary to know all the information about the optical circuit. Similar expressions can be obtained for the Mach–Zehnder interferometer. In particular, the issue of the influence of the interferometer coupler parameters on the Fourier amplitude was investigated in [26].

**Figure 3:**
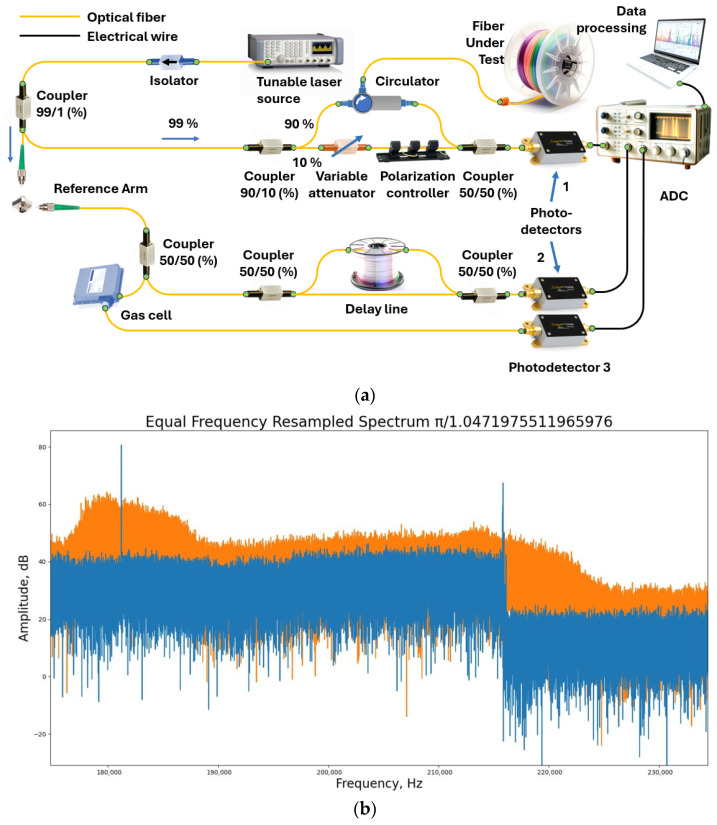
(**a**) OFDR setup based on Mach–Zehnder interferometer with two reference channels. (**b**) The output of this setup: without (orange) and with (blue) nonlinearity compensation. The orange plot represents the data obtained after FFT of the original signal. The blue plot shows the operation of one of the nonlinearity compensation algorithms, the data for which was obtained using an AUX. It is seen that the compensated data differ in that the beginning and end of the line are clear peaks; while in the raw data, rather wide areas can be observed instead of fiber ends, where information about the exact boundaries of the FUT is lost.

In the case of multiple reflectors, the Fourier transform series provides the longitudinal distribution of reflectors along the FUT. In the case of a continuous arrangement of reflectors/scatterers, each point of the fiber will correspond to its own frequency of the variable component of the intensity recorded via the photodetector. And, by dividing the total intensity acquired via the photodetector into periodic components, we will immediately obtain the spatial dependence of the backscattering coefficient, i.e., a trace. Thus, to obtain the spatial distribution of reflectors/scatterers in the fiber under test (FUT), we must apply the Fourier transform to the signal coming to the photodetector. In practice, the detected signal is not continuous, as discussed above, but discrete. In this case, the fast Fourier transform (FFT) is applied to the discrete signal to search for the “spatial spectrum”. In particular, this imposes serious requirements on the digitized photodetector signal: all samples must be equidistant in the frequency domain. In reality, this property is not met for most tunable OFDR lasers due to the nonlinear dependence of the optical frequency on time. In other words, expression (1) is not satisfied for them. The most common solution to this problem is direct measurement of the optical frequency using an auxiliary interferometer (AUX) with subsequent correction of the signal from the main interferometer. For absolute measurement of the optical frequency an absolute frequency standards – gas cells – are also added to the circuit. An example of the most complete circuit with the main and AUXs and a channel with a gas cell is shown in Figure 3a. Figure 3b shows two graphs that clearly illustrate the need, in an AUX, for the laser tuning nonlinearity compensation.

## 3. Basic Expressions Defining OFDR Parameters

The primary signal in OFDR systems is the digitized spectral transmission function of the main interferometer (time-domain signal) containing the FUT. However, in practical tasks, researchers are interested in the information contained in the traces obtained after applying FFT to this signal. The main parameters of traces include the maximum measurement range and spatial sampling. Below, what determines these two main parameters of the acquired traces will be considered. In this section, it will be assumed that all samples of the digitized signal are equidistant in the frequency domain, which allows FFT to be performed.

### 3.1. Maximum Measurement Range

It is easy to understand that the maximum coordinate on the trace corresponds to the highest-frequency modulation in the signal time domain. This means that the maximum range is determined by the minimum frequency discrete *δν*. Taking into account the properties of the Fourier transform, the expression for the maximum length is as follows:

(8) $$L_{\max}=\frac{\upsilon }{2\delta \nu }$$

where *v* is the speed of light in the fiber and the coefficient 2 in the denominator is due to Kotelnikov’s (Nyquist–Shannon sampling) theorem. As a rule, the value of *δν* is determined by the free spectral range of the AUX or the sampling frequency of the original signal *δν* = *γ*/*f_ADC_*. In the second case,

(9) $$L_{\max}=\frac{\upsilon f_{ADC}}{2\gamma }$$

In particular, it can be noted that increasing the signal sampling frequency *f_ADC_* facilitates the measurement of fiber lines at large distances. The same effect can be achieved by decreasing the scanning speed *γ*.

### 3.2. Spatial Sampling

The second main parameter of the trace is spatial sampling (by analogy with OTDR, the term spatial resolution is sometimes used)—it determines the smallest size of the recorded event. This parameter is extremely important, since it is high spatial resolution that is the main advantage of OFDR over other optical reflectometry methods. It is known that, in accordance with the properties of the Fourier transform, the sampling of the Fourier image is determined by the duration of the original signal. In relation to OFDR, this means that spatial sampling is determined by the width of one spectral scan ∆*ν* = |*ν*_max_ − *ν*_min_|, i.e.,

(10) $$\delta L=\frac{\upsilon }{2\Delta \nu }$$

It is worth noting that this parameter does not depend on the tuning speed. With equidistant digitization, the full scan consists of *N* = ∆*ν*/δ*ν* points. Then,

(11) $$\delta L=\frac{\upsilon }{2N\delta \nu }=\frac{L_{\max}}{N}$$

In other words, the maximum range and spatial sampling are related to each other through the number of samples in one scan of the original signal.

It is worth noting that it is quite difficult to simultaneously achieve high spatial resolution and a large measurement range. Moreover, in a number of works, the quality of the OFDR system is characterized by the ratio *L*_max_/*δL* = *N*. This means that it simply requires a large number of points per scan. Today, this parameter can reach a value to the order of 10^7^ [27].

## 4. Metrological OFDR Modifications

One of the main metrological parameters, which is also a kind of calling card of the OFDR method, is spatial resolution or spatial sampling.

Means of spatial-resolution improvement can be divided into two main categories. Firstly, software, such as when a new method of processing data obtained utilizing the same setup is used. And the second, hardware, which is when changes are made directly to the setup schematics or one of its parts. It is worth starting with software methods, since they allow one to use the information that is already contained in the raw data, and at the same time do not complicate or increase the cost of the system.

As already noted, spatial sampling is determined by the tuning range of the laser source. However, there are other limiting factors. The key one is the laser source frequency-tuning function nonlinearity. AUXs are used to compensate for this. Hence, the data they provide must be correctly processed.

The first thing worth mentioning is the equal frequency resampling (EFR) method used to compensate for a TLS nonlinear frequency sweep [28]. In this method, the authors propose to use the Hilbert transform to determine the instantaneous frequency of the AUX, instead of the previously used zero-crossing technique. This approach makes it possible to more correctly determine the instantaneous frequency and removes the limitation on the length of the delay line of the AUX, which, in the previous method, had to be four-times the desired length of the FUT. The use of EFR made it possible for OFDR to achieve a spatial resolution of the order of 10 µm for the first time.

But there are other effective ways to deal with the nonlinearity [27]. One of which, the authors call the periodic-phase-noise-estimated deskew filter (PPNE-deskew filter). The essence of the method is to pre-filter the signal from the AUX using a moving average and third-order Taylor expansion, and then “align” the signal from the main interferometer with it using the deskew filter. As a result of using such a technique, it was possible to achieve a spatial resolution of 500 μm over a measured length of 8 km, which, for the first time, yields a range-resolution ratio of more than 10^7^.

A synchronized wavelet transform is also used to compensate for the nonlinearity [29]. It is interesting that this study evaluates the instantaneous frequencies of a multicomponent signal. Despite the fact that the authors propose this method for linearization according to the usual scheme with an AUX, the demonstrated ability to determine the instantaneous frequencies of a multicomponent signal theoretically allows for linearization without an AUX but only using the signal from the main interferometer.

In addition to the development of software methods, the purpose of which is to more fully highlight useful information, in the field of OFDR, hardware methods are also being developed that help increase the amount of useful information in the raw data, make it more demonstrative, or even simplify the processing.

The greatest attention, as in the case of software methods, is allocated to the main distorting factor challenge—the nonlinearity of the radiation source tune. For example, the pre-distortion method is used for this, which can effectively suppress nonlinearity during the injection locking process [30]. As a result, a tunable radiation source was obtained with a scanning range of up to 60 GHz and a scanning speed of up to 15 THz/s and a high degree of tuning linearity. The OFDR scheme using this source was able, without subsequent software compensation for nonlinearity, to provide a spatial resolution of 1.71 mm for a measured line length of more than 2 km, which corresponds to the theoretical limit for the applied range and tuning speed. Moreover, the authors demonstrate successful determination of impacts on the FUT at distances of 1 and 2 km with spatial resolutions of 5 and 7 cm, respectively.

Other signal linearization methods are also demonstrated [31]. The idea is to install an electro-optical modulator after the radiation source and use it to influence the phase (and therefore the frequency of the signal). As a result, a tuning with a nonlinearity of 1.57% was obtained. This approach made it possible to achieve a spatial resolution of 8 cm with a measured length of about 2 km without software suppression of the nonlinearity of the tuning.

Spatial-resolution improvement methods are also proposed [32]. The authors consider replacing one continuous source tuning with several sequential and partially overlapping scans with a smaller range—a frequency comb. Due to the high linearity of the frequency comb, this method allowed achieving a spatial resolution of 1 mm without post-processing to suppress nonlinearity.

An important criterion for distributed studies using OFDR is the minimum level of back reflections that can be detected by the system. Thus, to provide a sensitivity on the order of −70 dBm and an output noise of ±4 mV, a compact low-noise photodetector based on a germanium–silicon photonic chip can be used as part of the OFDR setup [33]. It should also be noted that the results presented by the authors, for ease of comparison with other data, were obtained using zero-crossing. We believe that by using the EFR method, more outstanding results can be demonstrated with this detector.

Another OFDR improvement needed to increase the measurement range is an improvement in the signal-to-noise ratio (SNR). The required effect can be achieved, for example, by including a two-stage erbium-doped fiber amplifier (EDFA) into the setup [34]. The described approach, combined with the elimination of false reflections sources, made it possible to increase the SNR by 11 dB.

Also, SNR improvement can be achieved using correctly selected polarization of the interfering radiation [35]. The authors propose a way to challenge the constant change in polarization during the period of source tuning. By using a fixed 45° polarizer for the AUX and polarization-maintaining components for other part of the circuit, polarization fluctuations of less than 3° can be achieved. Compared to the previous adjustable polarizer design, this approach increases the SNR by 9 dB.

**Figure 4:**
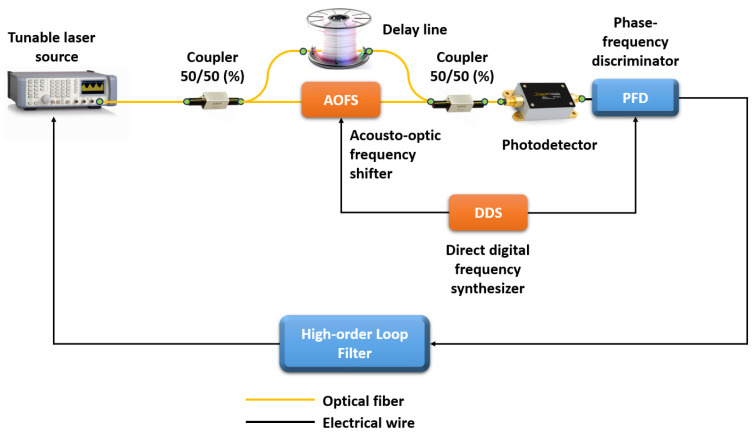
The laser stabilization proposed in [36].

Another important metrological parameter is the length of the FUT that OFDR can interrogate. In the case of studying short circuits or photonic integrated circuits, the line length can vary from several meters to several tens of meters. But there are cases when it is necessary to monitor a fiber communication line, study reflections, various inhomogeneities, and accurately measure the attenuation coefficient of an optical signal on a large segment of optical fiber. To solve this problem, an experimental setup was created that makes it possible to study long-length optical fibers using the OFDR technique (see Figure 4) [36].

High spatial resolution and accuracy, as well as the length of the measured line, are provided by a fiber laser with a high-order optical phase-locked loop. With innovations in OFDR design that compensate nonlinear frequency modulation of the laser, frequency sweep range and speed are optimized in addition to sweep linearization and improved time coherence. The authors’ experiments confirmed that centimeter-level spatial resolution is successfully achieved along the entire fiber communication line. The authors of the paper achieved a spatial resolution of ~3.2 cm and ~4.3 cm for a fiber line with a length of 130 km and 242 km, respectively. The accuracy of backscatter measurements was tested by the authors over the entire measurement range and was 0.5 dB. To improve system performance, the authors investigate OFDR vibration isolation issues and demonstrate successful solutions to these problems.

It is worth noting that the described metrology parameters are valuable not only for those who wish to study photonic and specialty fiber circuits. In many ways, such parameters determine the sensing characteristics of the system. In the next section, we will talk about these OFDR characteristics: we will look at how they are calculated and processed, and we will also look at the latest methods for improving them.

## 5. OFDR Sensing Modifications

### 5.1. Basic Principles of Sensing Using OFDR

**Figure 5:**
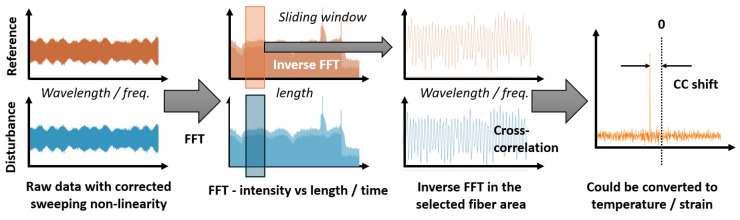
Standard algorithm for obtaining temperatures and strains in OFDR.

To discuss the latest advances in sensing based on OFDR, it is necessary to briefly recall the basic principles by which various physical quantities can be measured along the length of a sensor optical fiber. First, let us imagine that we have only one contrast sensor in the line, which is a fiber Bragg grating (FBG), the operating principles of which are well described in the literature [37,38,39]. Its resonant wavelength is determined with the following equation [40]:

(12) $$\lambda_{res}=2n_{eff}\Lambda$$

where *n_eff_* is the effective refractive index; Λ is the period of the FBG. Looking at Equation (12), it is easy to see that there is a linear relationship between period and wavelength. An optical fiber with its inherent inhomogeneities caused by conglomerates of germanium oxide and silicon oxide molecules is essentially a weak FBG, the spectral shift of which can be easily detected upon impact. The standard method of interrogating such an array will not allow the responses from individual parts of the fiber to be separated. This is where OFDR technology comes to the rescue. Soller, Froggatt, Gifford, and co-authors, who conducted their research at Luna Innovations, were pioneers in this area [41,42,43]. They showed that all physical quantities possible for this method are obtained by detecting changes in the refractive index along the length of the optical fiber. They are fully characterized by the phase of backscattered radiation that changes when the optical fiber undergoes an impact. This information can be extracted from backscattering data in various ways. A kind of gold standard is a method based on calculating cross-correlation functions (CCFs) of Rayleigh backscattering spectra (RBS) before the impact to an optical fiber and during it. In general, the algorithm for calculating the signal phase at each point is quite simple. First, the original data, corrected by an AUX or other method of frequency-tuning nonlinearity compensation, are subjected to an FFT, during which the amplitude and phase spectrum are obtained. The phase spectrum cannot be immediately used to reconstruct the phase for each beat frequency, since it is distorted by various noises. Therefore, the inverse windowed Fourier transform is then calculated for the impacted and still fiber:

(13) $$I_{z}(t,\nu )=\Phi_{\nu }^{-1}\{{\left[R(t,x)\right]}_{z}\}$$

where *z* is the size of the window in counts; *R* is the intensity value at a spectrum point with coordinate *x* at a time *t*; *ν* is the optical frequency; $\Phi_{\nu }^{-1}${·} is the inverse Fourier transform. After this, a normalized CCF is calculated for the reference and measurement spectrum, which has a characteristic peak:

(14) $$\Psi_{z}(t_{a},t_{r})=\frac{{I^{\prime }}_{z}(t_{d},\nu )}{S({I^{\prime }}_{z}(t_{d},\nu ))}⊗\frac{{I^{\prime }}_{z}(t_{r},\nu )}{S({I^{\prime }}_{z}(t_{r},\nu ))},$$

where ⊗ stands for the convolution; the subscripts *d* and *r* denote “disturbance” and “reference”, respectively; *S*(...) is the standard deviation; ${I^{\prime }}_{z}(t_{d},\nu )=I_{z}(t_{d},\nu )-{\bar{I}}_{z}(t_{d},\nu ),$ where ${\bar{I}}_{z}(t_{d},\nu )$ is the average value of the data array $I_{z}(t_{d},\nu )$; ${I^{\prime }}_{z}(t_{r},\nu )=I_{z}(t_{r},\nu )-{\bar{I}}_{z}(t_{r},\nu ),$ where ${\bar{I}}_{z}(t_{r},\nu )$ is the average value of the data array $I_{z}(t_{r},\nu )$. The frequency coordinate of this peak is expressed as follows:

(15) $$\Omega_{r}^{d}=find\max(\Psi_{z}(t_{d},t_{r})),$$

where *find*max returns the maximum value argument of the function Ψ*_z_*(*t_d_*,*t_r_*). The value $\Omega_{r}^{d}$ is proportional to the phase difference of the signal Δ*ϕ*, as well as to the temperatures and strains acting on the optical fiber at a specific position:

(16) $$\Omega_{r}^{d}=K_{\varepsilon }\Delta \varepsilon_{r}^{d}+K_{T}\Delta T_{r}^{d}$$

where *K_ε_* and *K_T_* are proportionality coefficients for strains and temperatures, respectively. This entire process is shown schematically in [44], and an adapted version is presented in Figure 5.

Despite the fact that this approach is widely used in commercial devices, it is not deprived with various disadvantages: narrow ranges of temperatures and deformations, false alarms due to phantom bursts in the correlation function, time and resource consuming calculations, etc. The rest of this section will show various improvements and alternatives to this approach, as well as various hardware modifications—including the use of custom fiber sensor designs. It is with such sensors that we will begin to consider various OFDR improvements for distributed fiber sensing.

### 5.2. OFDR Parameters Enhancing with Sensing Fibers

The problem of improving sensing characteristics is the one inherent to the distributed sensor systems based on different principles. Sensors based on OFDR are no exception. One of the significant limitations is the low SNR of the data. In this case, an approach that has already been tested in the field of distributed acoustic sensors is used [45,46,47]. This approach uses backscatter enhanced optical fibers (BEOF). In the OFDR, BEOF is utilized for fast and highly precise measurement of large deformations [48]. The authors show that by increasing the RBS intensity, the SNR grows, resulting in a clearer and more readable CCF. The authors also show a fast algorithm for adaptive local feature extraction and a matching algorithm, which replaces the well-known cross-correlation method for obtaining strain or temperature. Since the optical fiber used by the authors contained a distributed weak FBG at a fixed wavelength, part of the scattering spectrum had a burst characteristic of Fresnel reflection, the extraction of the frequency coordinate of which requires less complex and computationally expensive approaches. The authors tested the effectiveness of their algorithm. The test showed that the exposure extraction time is about 25% of the time of the known approach.

BEOF can also be used for distributed temperature measurement [49]. Here, the highly scattering locations were randomly distributed in the fiber, contrary to the previously described sensor. A standard cross-correlation algorithm was used for impact extraction. The temperature sensitivity coefficient was 7.814 µm/(m°C), the average relative temperature measurement error was 1.12%. In this case, the event positioning error was only 0.02 m for the fiber segment affected by temperature.

High-temperature OFDR measurements are also possible using an annealed ZrO_2_-doped optical fiber with a 40.5 dB amplified scattering signal [50]. The zirconia-doped fiber has an initial loss of 2.8 dB/m and low signal drift at 800 °C. Zirconium dioxide-doped fiber can be used as an alternative to a distributed nanograting sensor. Both of these approaches enable distributed OFDR measurements over several meters with 1 cm spatial resolution at high temperatures.

The sensing capabilities of special fibers are truly impressive, but their use is associated with various difficulties. First, existing optical fiber communication cables or fibers previously integrated into the facility are sometimes used to monitor physical quantities. Most often these are the widely used telecommunication fibers SMF-28 or similar ones. Secondly, complex sensing fibers are usually much more expensive than conventional ones. This is due both to the addition of extra doping elements at the stage of the MCVD process, and to the creation of reflectors in the already fabricated fiber. Third, a significant limitation is the attenuation coefficient of the optical fiber signal, which usually exceeds standard values for these types of fibers. Enhanced Rayleigh scattering can, in some cases, significantly limit the sensor length. In such cases, it is appropriate to seek a reasonable compromise between sensing parameters and FUT length. That is why researchers often focus their attention on software signal processing, which makes it possible to significantly improve the sensing characteristics of systems.

### 5.3. OFDR Software Improvements

A logical and obvious way to exploit the redundant data in RBS arrays arranged sequentially along a time or spatial scale is via algorithms similar to digital image processing methods. In essence, such data are two-dimensional arrays in which it makes no sense to limit mathematical operations only to columns or only to rows. This approach has been successfully applied in the abovementioned distributed acoustic sensors [51,52,53] and systems based on Brillouin scattering [54,55,56]. Since the RBS set has quite a lot in common with the Brillouin gain spectra (BGS) set, the principles for processing such data may be somewhat similar.

For example, OFDR performance is experimentally studied using image processing techniques including wavelet transform and Gaussian filter arithmetic [57]. All these approaches have been applied to increase the range of strain measurements. RBS are considered as a two-dimensional image, which is processed using the algorithms mentioned above, and after that, the frequency offset is determined. Compared with the traditional processing method, the approach proposed by the authors is able to suppress phantom peaks caused by the spatial distortion of the stretched fiber. Strain gradients up to 7000 με can be recorded by the proposed methods with a spatial resolution of 4 mm.

**Figure 6:**
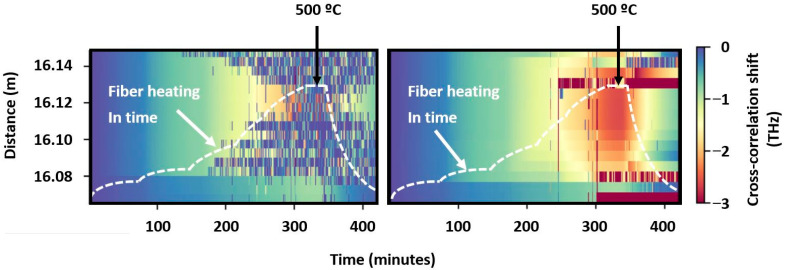
Evolution of the temperature field obtained via OFDR means without the algorithm proposed by Sweeney and Petry (**left**) and with it (**right**).

Sweeney and Petrie also perform two-dimensional analysis of backscatter data but use data not from spectra adjacent in the spatial coordinate of the fiber but from the time evolution of the same ones [58]. This allows one to obtain a better CCF with a clearly localized peak without any phantom bursts. The efficiency of the algorithm is clearly shown in Figure 6.

From the left part of the figure, it can be seen that at approximately 110 min of warming up, the CCF, calculated in the standard way, is already processed incorrectly, since the maximum is erroneously provided at zero. The operation of the algorithm proposed by the authors is characterized by the right part of the figure. When using this algorithm, temperature calculating errors become much smaller; incorrect values appear closer to extreme heating. In some locations of the temperature field, the algorithm incorrectly produces the maximum possible shift of the correlation function, but still there are much fewer bad fragments in the figure on the right.

The distributed OFDR-based fast-changing strain sensor also uses machine learning methods to estimate the radiation phase [59]. Notably, the neural network was trained using a simulated set of optical signals reconstructed from the Rayleigh scattering characteristics of a fiber subjected to strain. Next, the efficiency of the neural network was verified by the authors using another set of numerically generated data. At this stage, the achieved accuracy of strain detection was also compared with the standard homodyne detection method. At the final stage, the approach demonstrated by the authors of this work was tested in real experimental measurements. These experiments showed an improvement in the SNR of the spectrogram in the distributed acoustic sensor mode by at least 5.1 dB compared to the standard approach.

One-dimensional data processing methods are also often very effective [60]. Here the authors use interpolation of the RBS in OFDR. This work examines the effectiveness of various interpolation algorithms to improve the accuracy of CCF peak position determination. As a result, these measures lead to a reduction in the deformation determining error. The authors show that interpolation by adding zeros on one side of a sliding window in the spatial domain (just before the inverse FFT) provides accuracy gains without reducing spatial resolution. In this simple way, it is possible to record deformations of 3 με with a fiber coordinate accuracy of 1 cm over a sensor length of 21.4 m, while the measurement error is 3.3 με.

**Figure 7:**
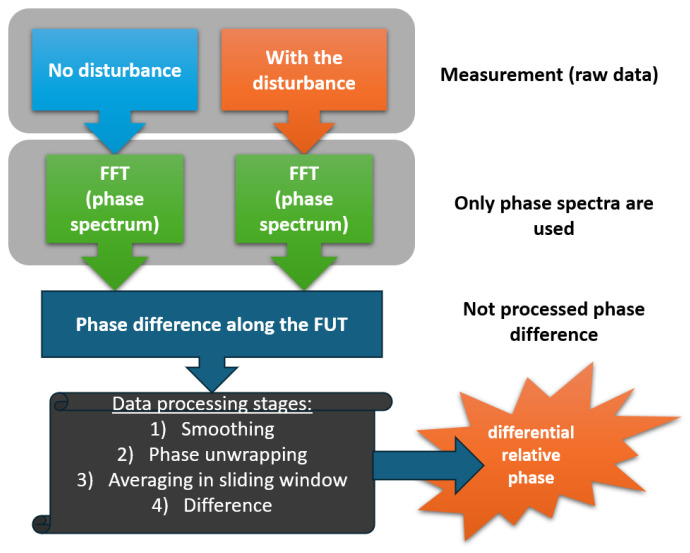
The algorithm proposed in [61].

Wang et al. demonstrate a new algorithmic approach for static strain measurement using OFDR [61]. By performing two separate measurements (with and without stimulation), it is possible to achieve a distributed strain measurement by obtaining the differential phase between the two states, which can significantly reduce the calculation time compared to the correlation method and increase the accuracy of the measurements. The phases of the two signals are as follows:

(17) $$\phi_{1}=2\pi \tau_{1}f_{01}+\varphi_{1}(\tau_{1}),$$

(18) $$\phi_{2}=2\pi \tau_{2}f_{02}+\varphi_{2}(\tau_{2}),$$

where *φ*_1_(*τ*_1_) and *φ*_2_(*τ*_2_) are the phase of backscattered radiation for the reference state (without influence) and the measurement state (with influence), respectively. *f*_01_ and *f*_02_ are the initial optical frequencies for these two states. *δf* = *f*_02_ − *f*_01_ is the initial difference between the optical frequencies of the reference state and the measurement state. *φ*_1_(*τ*_1_) and *φ*_2_(*τ*_2_) are defined in [61] as the residual nonlinear phase in the spatial domain. The reasons for the appearance of *δf* are the error in determining the starting wavelength in TLS and the digitization error. *τ*_1_ and *τ*_2_ are the radiation time delays for the reference state and for the measurement state, which can be expressed as follows:

(19) $$\tau_{1}=\frac{2n_{1}(z)z}{c},$$

(20) $$\tau_{1}=\frac{2n_{2}(z)[z+\Delta z]}{c},$$

where *c* is the speed of light in a vacuum, and *n*_1_(z) and *n*_2_(z) are the distributions of the refractive index along the FUT before and during impact, respectively. *δn*(*z*) = *n*_2_(*z*) − *n*_1_(*z*) is the difference in the refractive indices of the fiber core in these states. Next, from (17) to (20) the following is obtained [61]:

(21) $$\frac{d\phi (z)}{dz}=-\frac{4\pi }{c}\left[\delta fn_{1}+f_{01}\delta n(z)+f_{01}n_{1}(z)\frac{d\Delta (z)}{dz}\right]+\frac{\Delta \varphi (z)}{dz},$$

where $\frac{d\Delta (z)}{dz}$ is the longitudinal mechanical stress in the fiber sensor. The last term in this equation is the noise component that needs to be eliminated for high-precision recording of deformations or temperatures along the sensor. To do this, the authors of the work in question created a special algorithm that includes phase matching and noise reduction modules. As mentioned above, to extract the phase of both states (with and without perturbation) it was not necessary to calculate the inverse Fourier transform and the correlation function, since the emission phase value was extracted directly from the phase spectrum of the backscattered signal. A simplified diagram of the algorithm proposed by the authors is shown in Figure 7.

As in the works mentioned at the beginning of the section, the OFDR system presented by the authors used a “fully grated” optical fiber as the FUT, which has stronger backscattering than standard SMF fibers. In this case, the setup used by the authors can be considered standard. In their experiments, the static strain range was 0 to 25 με, the spatial resolution in sensing mode was 4.35 cm, and the minimum detectable strain change was 0.25 με. The total fiber length was 36 m. A similar signal processing technique, but with its own algorithmic features, is also demonstrated by Zhao et al. [62].

An interesting group of studies is aimed at increasing the length of the sensor using software methods. In [63], the authors demonstrated the use of an original technique for radiation coherence restoring based on phase-noise compensation. The idea behind this approach is to numerically generate a series of new reference signals such that they explicitly contain the phase noise integrated into the signal at a given sensor location. The result shows an increase in the RBS correlation coefficient to values acceptable for sensors at a line length of about 1 km.

A spectral segmentation method for distributed measurement of impacts over line lengths greater than a kilometer is described in [64]. Based on the traditional cross-correlation demodulation method, the proposed method divided the overall signal into equal time segments, treating each such segment as an independent measurement. Next, the spatial distribution is corrected via correlation comparison of individual segments with the full signal. Segmentation has been shown to effectively suppress phase noise that accumulates over a large tuning range at long distances, and thus expands the tuning range of the laser that can be effectively used. Spatial pre-correction refines spatial-domain uncertainty caused due to segmentation, which reduces the error from 10 m to millimeters, allowing precise splicing of spectra and expanding the spectral range, thereby increasing the range of measured deformation. The reported experimental results demonstrate a strain sensitivity of ±3.2 με (3σ) over a length of 1 km with a spatial resolution of 1 cm and an extended strain measurement range of up to 10,000 με.

Using OFDR, temperature and strain perturbations were detected in a 3 km fiber loop of polarization-maintaining anisotropic optical fiber [65]. The authors proposed a method for deviation compensation in the distribution of reflective events under the influence of temperature and deformation. The main interferometer was made of polarization-maintaining fiber. Radiation was injected into the circuit at an angle of 45 degrees to the polarization axes. With uniform heating of the loop, a spatial resolution of 5 cm for impact determination and a deformation measurement accuracy of 1.2 με were demonstrated.

There are also approaches that provide filtering not at the processing stage of scattering spectra or CCFs but at later ones [25]. It is shown that for some specific applications it is sufficient to use simple filtering in the spatial domain. Due to parasitic reflections in the FUT, including reflections from the far end of the fiber, phantom peaks quite often appear on the trace, which have nothing to do with the physical nature of scattering or the impact on the sensor. In applications such as object shape reconstruction using OFDR, such phantom peaks distort the signal. Fortunately, in this case, sudden changes in temperature and deformation are almost impossible. This makes it possible to apply filtering based on knowledge about the object being monitored. The results of experiments conducted by the authors showed that the shape reconstruction error decreases significantly with a sensor length of 0.5 m.

The recent work of Zhang et al. [66] cannot be ignored, in which the authors made successful attempts to improve not just one characteristic of the sensing system but to improve them simultaneously. The authors used a standard OFDR setup, and a feature of their approach was the use of a special artificial neural network, which was trained on noise data generated taking into account the physical aspects of the OFDR functioning. When generating the data, they assumed that strain changes occur in the range from −10,000 to 10,000 με. Using this approach made it possible to obtain a very impressive set of sensor characteristics of the system: a sub-millimeter spatial resolution of 0.857 mm, an impact determination accuracy of 0.91 με, and a sensor line length of 140 m.

### 5.4. OFDR Hardware Modification to Improve Sensing Performance

**Figure 8:**
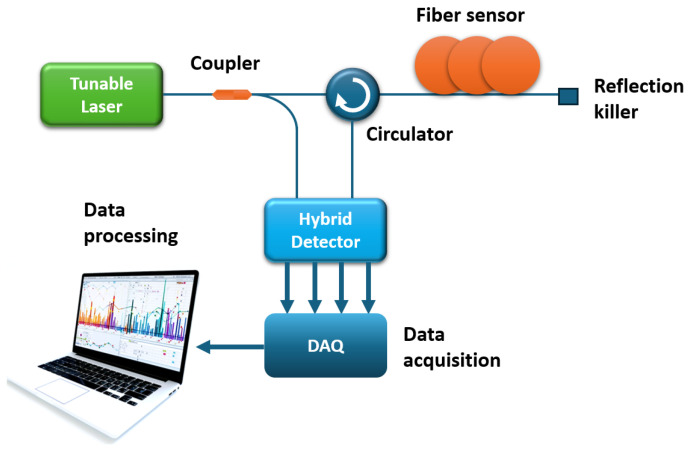
The setup proposed in [67].

Despite the fact that OFDR setups recently proposed by various research groups are almost identical, and only the signal processing approach changes, there are cases in which the main measures to improve sensor parameters are implemented using hardware modifications. One of such cases is reported by Jderu et al. [67] (including Marcelo A. Soto). The experimental setup presented by the authors is depicted in Figure 8.

It is easy to see that in the setup, interference of the reference signal and backscattered radiation occurs in a hybrid detector with polarization diversity. This detector has four outputs: IS, QS, IP, and QP. Where I and Q are 90° apart in phase, and S and P are 90° apart in polarization. The authors claim that this approach provides a higher-quality sensor signal, separated by two polarizations, compared to the use of a conventional polarization beam splitter (PBS). The signal processing method used by the authors is practically no different from that presented in Figure 5. The only difference is the fact that the four channels of the hybrid detector must first be converted into one channel. The authors use simple arithmetic operations, adding information about two channels separated in phase into one channel (for each polarization) and then calculating the vector sum:

(22) $$P=\sqrt{(IS)+QS^{2}+{\left(IP+QP\right)}^{2}}$$

Without averaging and with a data acquisition time of 230 ms, the authors’ preliminary results show a spatial resolution of 5 cm and a temperature resolution of about 0.1 K on a 3 m-long fiber sensor.

**Figure 9:**
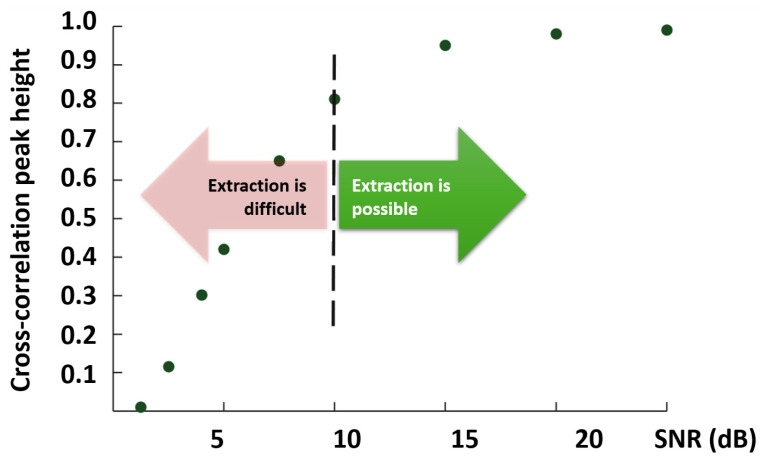
Estimation of the possibility of temperatures and strains extracting from the SNR of the real part of the OFDR spectrum.

Another hardware modification to improve the sensor properties of the system was made in the work already mentioned in this review. The authors [34] used two-stage EDFA located directly in front of the photodetector. As already noted in the previous section of our review, this allows you to increase the dynamic range of the system and increase the SNR and probing range without disturbing the useful interference signal. This will make it possible to study longer optical fibers. However, it should be noted that the ability to extract temperatures and strains also depends on the SNR. On a regular OFDR trace, we see a picture of reflections; it represents the real part of the spectrum. By measuring the SNR over the real part of the spectrum, we can estimate the SNR of the phase part of the spectrum containing information about the influences. Thus, the authors of the work [68] presented a graph with which one can evaluate the possibility of extracting the impact based on the SNR of the real part of the spectrum (Figure 9).

The authors claim that temperature and strain extraction is possible starting from when the SNR = 10 dB. Also, optimization of the SNR in OFDR is possible by integrating polarization controllers and attenuators into the arms of interferometers [28,69,70].

Yang et al. demonstrate a new approach to increase the strain measurement range of OFDR [71]. They demonstrate the principle of dual-frequency phase-sensitive OFDR, which ensures sufficient stability of the two frequencies and, as a consequence, stability of the synthesized lower frequency. Artificially increasing the wavelength allows one to achieve more convenient phase differences for obtaining information about the effect on the fiber sensor. The theoretical limit for increasing the dynamic range in comparison with a standard system based on an OFDR, according to the authors, is a gain of approximately 193 times.

Some hardware modifications make it possible to increase the length of the sensor line. Thus, in the system proposed by the authors with internal modulation, a laser on a silicon photonic crystal is used as a source of laser radiation [18]. Frequency tuning in the range up to 10 GHz is achieved by controlling the output voltage of the signal generator, which provokes a temperature change in the external laser cavity. The complexity of the proposed internal modulation system is much lower than that of the traditional external modulation OFDR system, which uses a narrowband laser and an SSB modulator to tune the wavelength. Cross-correlation analysis is used by the authors as a sensing mechanism to assess the similarity between Rayleigh scattering signals and to achieve event localization. The authors managed to achieve a sensor length of more than 100 km, and the system was tested with rapidly changing deformations (vibrations) of the sensor. The spatial resolution of the obtained data was tens of meters (43.0 and 16.8 m for external and internal modulation, respectively).

Experience in OFDR using a fiber laser source with a phase-locked loop circuit is demonstrated [72]. The frequency-switching fiber laser was synchronized with a Mach–Zehnder interferometer to suppress tuning nonlinearity and enhance laser coherence, resulting in highly coherent linear tuning over a 1 GHz bandwidth in 25 ms. The authors showed a decrease in the spectral width of the laser line from 5 kHz to 1.4 Hz with an averaging time of 2 s. As a result, a spatial resolution of 10 cm was obtained on a 20 km-long sensor with a tuning time of 25 ms in the 200 MHz range and a spatial resolution of 72 cm on a 20 km-long sensor with a tuning time of 5 ms.

### 5.5. Comprehensive Measures to Improve OFDR Sensing Performance

**Figure 10:**
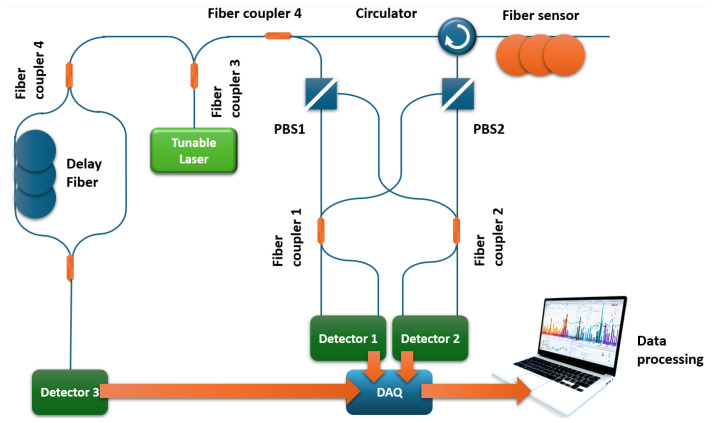
OFDR schematics presented in [73].

An interesting complex solution combining hardware modifications and signal processing using artificial intelligence was presented recently [73]. Firstly, it is worth noting that they demonstrated an original technical solution that makes it possible to reduce the influence of polarization fluctuations in the sensor. Instead of using a coupler, in which an interference between incident and backscatter signals occurs in a traditional OFDR, they used two polarization beam splitters (PBSs) and two couplers connected as shown in Figure 10. This scheme has been used by various authors before; however, it is not so typical for popular OFDR designs.

**Figure 11:**
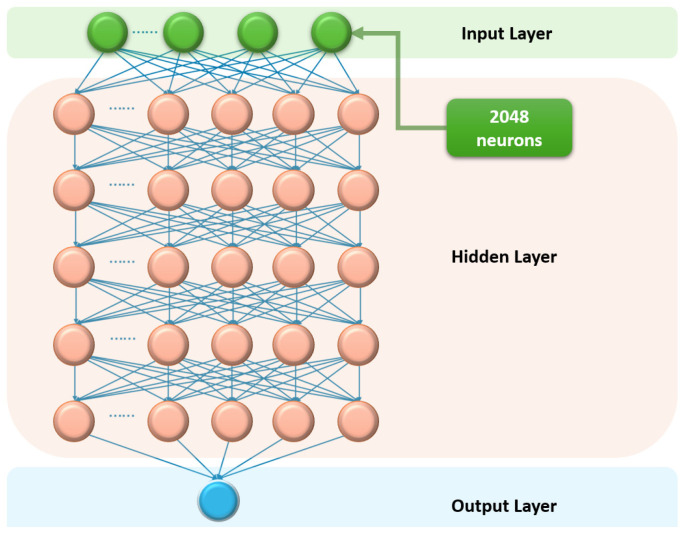
Multilayer perceptron used in [73] to improve OFDR sensing performance.

The second important difference was the use of a neural network algorithm instead of the cross-correlation method. To obtain impact data, the authors chose a multilayer perceptron based on a backpropagation algorithm. The multilayer perceptron is a well-known, traditional, fully connected neural network. Its schematic representation is provided in Figure 11. The original RBS were sent to the 2048 neurons of the input layer both during training and during application of the algorithm. In addition to the hidden layers, where the number of neurons depends on the specific hardware settings and application; the network structure contains a single output neuron that outputs temperature or strain values. This algorithm operates successfully even when the overlap between spectra with and without impact is very insignificant.

The RBS training data were generated by artificially shifting the spectra without impact and adding additional ones. The authors consistently optimized the training and activation functions, as well as the number of perceptron layers. As a result, a perceptron with five hidden layers with an average classification accuracy of up to 90% was obtained. Compared with the traditional correlation algorithm, the neural network provided a larger measurement range and higher measurement accuracy and requires fewer computing resources and processing time.

Worthy of attention is the publication by Li et al. [74], in which the authors presented an OFDR setup very similar to the one described above. The entire main interferometer is implemented using anisotropic optical fibers and polarization-maintaining components. This made it possible, with proper signal processing, to significantly improve the quality of the obtained data and to carry out sensor measurements on a 1.5 km-long fiber with a spatial resolution of 5 cm, while the temperature measurement error was ±0.2 °C.

**Figure 12:**
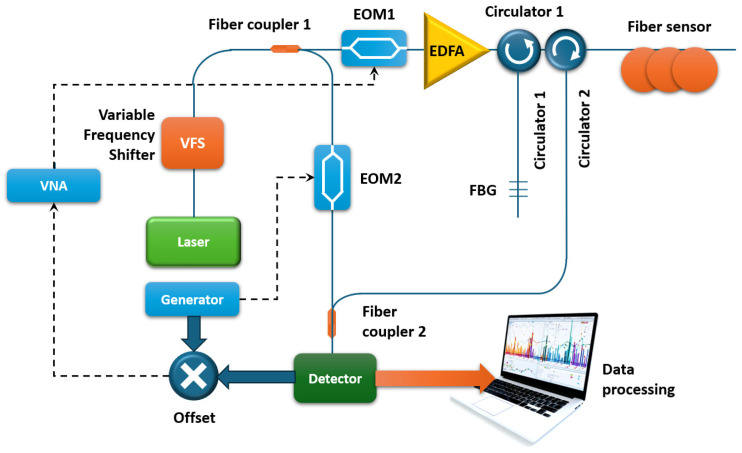
OFDR using vector network analyzer (VNA) proposed in [78].

An interesting variant of hardware modification is the creation of an OFDR based on a radiation source operating at a single wavelength. This approach is usually used when the radiation of a single-frequency laser, which does not change the wavelength, is used in Brillouin reflectometry and Brillouin analysis systems [75,76,77]. Such a configuration, but for OFDR, was proposed in [78]. Figure 12 shows the experimental setup proposed by the authors.

The setup is equipped with an electro-optical modulator operating in the SSB (single-sideband modulator) mode, controlled via an RF oscillator. The SSB modulator receives the laser radiation directly. The frequency shifting by means of the SSB is necessary for scanning the laser frequency and, hence, obtaining the RBS. Thus, the radiation from a single-frequency source at a wavelength of 1550 nm was first-frequency shifted. Then the frequency-shifted light was split by means of a fiber coupler in a 90/10 ratio to send the probe light to the horizontal (Figure 12) branch and the local oscillator (vertical) branch. The signal in the first branch was intensity modulated by the electro-optical modulator (EOM1), shifted at its zero point, and controlled by means of VNA; then, it was amplified by means of EDFA. The authors tested their setup in the single- and dual-side harmonic (SSB and DSB) modes, respectively. Since such frequency scanning approaches did not allow for wide frequency variation, the resolution of the measurements obtained was limited to 3 cm. However, with this resolution, the authors were able to obtain a temperature extraction accuracy of about 50 mK.

In another paper [79], the authors proposed a method of complex noise suppression to achieve the highest OFDR resolution. In this method, the corrected radiation phase data were used to construct a complex signal, and then both the real and imaginary parts were cleared of noise using wavelet analysis. Two arrays of cleared signals were reconstructed to a complex form, which allows obtaining the correct phase values. Also, the spatial position correction algorithm solves the problem of phase drift due to the accumulation of distortions introduced via deformations. In addition, the authors used high numerical aperture fiber to increase the Rayleigh scattering intensity by 15 dB. This approach yielded the authors impressive results: a spatial resolution of 0.89 mm, a root mean square error in deformation determination of 1.5 µε, and an upper limit of the deformation range of 2050 µε.

**Figure 13:**
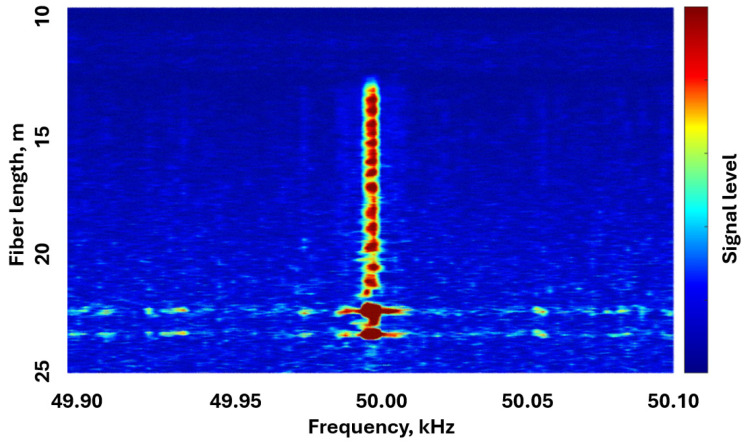
Results obtained in [82] for OFDR when detecting a vibration.

Another important improvement of the system is the increase in the measurement speed. Some research groups have managed to increase it so that it is possible to detect vibrations, i.e., very fast deformations (but often with less accuracy than with standard measurement). In previous years, numerous authors have obtained different vibration characteristics using OFDR. Thus, in [80], the detection of kilohertz vibrations on a fiber length of 950 m with a spatial resolution of 12 cm is described. In [81], the authors use sinusoidal frequency tuning of the radiation source and obtain detection ability for 400 Hz at 64 km with a spatial resolution of less than 10 m. But the study in which L. Marcon, A. Galtarossa, and L. Palmieri [82] actually created a full-fledged distributed acoustic sensor with high resolution seems to be one of the most original. Their results are shown in Figure 13.

This system also underwent comprehensive modification of both the hardware and software. The authors thoroughly investigated the OFDR phase noise and developed an algorithm for its compensation. First, OFDR traces were obtained in the frequency domain under vibration load and in a normal state. To solve the problem of nonlinearity of the coordinate axis after tuning-errors compensation using an AUX, the authors integrated a source of sound vibrations with a frequency of 3 kHz into the hardware of the distributed acoustic sensor at specified locations. This helped the research team collect data to eliminate distortions and build more accurate RBS using the oversampling method. The practical results obtained for a FUT length of 25 m are truly impressive: a frequency of 50 kHz was detected quite confidently and spatially localized with an accuracy of up to tens of centimeters, which is an impressive indicator even by the standards of distributed acoustic sensors based on the φ-OTDR principle.

## 6. Discrimination of the Temperature and Strain

Since the OFDR outputs a shift in the correlation function in the sensor mode, which can be related to both temperatures and deformations, it is unclear which physical factors, and to what extent, contributed to the changes in this value. In this case, only one parameter is measured, but to obtain two physical quantities, two parameters must be obtained. Among the first to solve this problem were the same researchers from Luna Innovations, whose work resulted in a patent [83], describing the process of discrimination of temperature and deformations using an anisotropic optical fiber used as a sensor. We will analyze this patent in particular detail.

So, imagine that two polarization modes are reflected at the same location of a fiber sensor. Of course, these reflections occur at different wavelengths, since the different polarization axes have different refractive indices. Consequently, the RBS from the two polarization modes are shifted relative to each other. Although the RBS at any location of an anisotropic fiber is random, the spectral shift between the two modes can be detected by calculating the autocorrelation function (ACF).

**Figure 14:**
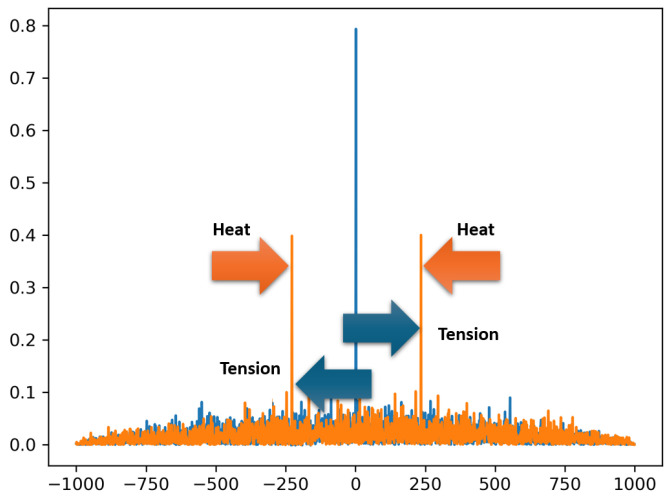
ACF presented in [83].

The ACF of the spectrum for a particular location of an anisotropic fiber is shown in Figure 14. In this figure, one can see a pair of peaks at a certain distance from the central peak. The central peak is a normal component of the ACF (shown as a blue line). The side peaks (shown as an orange line) are the result of a shifted copy of the RBS pattern. The number of discrete samples between the central peak and any of the side peaks is calculated by a threshold or any other suitable algorithm, and this value characterizes the spectral shift between the two polarization modes. Since the birefringence of an anisotropic fiber depends on temperature, when a given location of the fiber sensor is heated, these peaks shift closer to the central peak, as shown in the figure. In case the anisotropic fiber sensor is subjected to stretching, these side peaks will move away from the center.

If necessary, the ACF can be interpolated to obtain a larger number of points and, therefore, increase the resolution. The authors of the patent also note that for each section of the fiber, the light signal intensity as a function of frequency/wavelength will be obtained for both s and p polarization modes in the anisotropic fiber sensor. The measured light intensities for the s and p polarization modes are linearized using data from the AUX, as in most other OFDRs. Then, as in the typical processing procedure, the linearized s and p light intensity data are subjected to a complex Fourier transform, which will provide a relationship between the obtained scattering parameters and the coordinate in the fiber sensor. The OFDR trace, as well as the imaginary part of the spectrum, are stored in the system for further mathematical processing. Complex numbers are used from the obtained data set, characterizing the s and p polarization states, which correspond to a certain location in the fiber sensor. The vector sum of two amplitude arrays s and p, obtained after the Fourier transform, is calculated to form a single amplitude spectrum. The authors of the patent separately dwell on the subtraction of the average RBS value, which does not carry useful information, but introduces distortions in the subsequent calculation of autocorrelation.

After this, the authors resort to calculating the ACF, which gives rise to a central, zero autocorrelation peak and two side autocorrelation peaks for the polarization modes s and p.

To extract the rest of the information, the already mentioned CCF is used, which is calculated for two measurements: in the unloaded state and with a load. This procedure is no different from that already described in the previous section of this review. Then, the obtained data are combined in a system of equations, the solution of which ensures a satisfactory discrimination of deformation and thermal effects.

First, a calibration matrix is constructed based on the results of the calibration experiment:

(23) $$\left[\begin{array}{l} \Delta \upsilon_{auto}\\ \Delta \upsilon_{cross} \end{array}\right]=\left[\begin{array}{l} \zeta_{Ta}\text{​}\;\;\;\;\zeta_{Tc}\\ \zeta_{\varepsilon a}\;\;\;\;\zeta_{\varepsilon c} \end{array}\right]\left[\begin{array}{l} \Delta T\\ \Delta \varepsilon \end{array}\right],$$

where $\Delta \upsilon_{auto}$ and $\Delta \upsilon_{cross}$ are the autocorrelation and cross-correlation shifts, Δ*T* and Δ*ε* are the temperature and strain changes, respectively, and $\zeta_{Ta},$$\zeta_{Tc},$ $\zeta_{\varepsilon a}$ and $\zeta_{\varepsilon c}$ represent the proportionality constants between these parameters. Once the parameters $\left[\begin{array}{l} \zeta_{Ta}\text{​}\;\;\;\;\zeta_{Tc}\\ \zeta_{\varepsilon a}\;\;\;\;\zeta_{\varepsilon c} \end{array}\right]$ are obtained, measurements can be made and the values $\left[\begin{array}{l} \Delta T\\ \Delta \varepsilon \end{array}\right]$ can be obtained:

(24) $$\left[\begin{array}{l} \Delta T\\ \Delta \varepsilon \end{array}\right]=\frac{\left[\begin{array}{l} \Delta \upsilon_{auto}\\ \Delta \upsilon_{cross} \end{array}\right]}{\left[\begin{array}{l} \zeta_{Ta}\text{​}\;\;\;\;\zeta_{Tc}\\ \zeta_{\varepsilon a}\;\;\;\;\zeta_{\varepsilon c} \end{array}\right]}=\frac{\left[\begin{array}{l} \zeta_{\varepsilon c}\text{​}\;\;\;\;-\zeta_{Tc}\\ -\zeta_{\varepsilon a}\;\;\;\;\zeta_{Ta} \end{array}\right]\left[\begin{array}{l} \Delta \upsilon_{auto}\\ \Delta \upsilon_{cross} \end{array}\right]}{\zeta_{Ta}\zeta_{\varepsilon c}-\zeta_{\varepsilon a}\zeta_{Tc}}$$

Then, the deformations and temperatures are expressed as follows:

(25) $$\Delta \varepsilon =\frac{\zeta_{Ta}\Delta \upsilon_{cross}-\zeta_{\varepsilon a}\Delta \upsilon_{auto}}{\zeta_{Ta}\zeta_{\varepsilon c}-\zeta_{\varepsilon a}\zeta_{Tc}}$$

(26) $$\Delta T=\frac{\zeta_{\varepsilon c}\Delta \upsilon_{auto}-\zeta_{Tc}\Delta \upsilon_{cross}}{\zeta_{Ta}\zeta_{\varepsilon c}-\zeta_{\varepsilon a}\zeta_{Tc}}$$

Of course, for the system of Equation (23) to have certain solutions, the proportionality coefficients must differ to some extent. The reason why the proportionality coefficients for strain and temperature are different can be seen by observing the behavior of the ACF during various effects on the fiber sensor. Thus, the peaks of the ACF show how the two polarization modes behave. The authors of the patent remind that the separation of the radiation into two polarization modes is caused by the presence of special loading borosilicate rods, which are located near the core of the fiber sensor. During the production of optical fiber, when the preform is melted, the stresses in the cross section of the fiber are zero. But when the fiber cools, the loading rods are compressed at a different rate than the surrounding glass, and stresses are created in the material. These stresses are what separate the spectral components of the two polarization modes. When the glass is heated, such discrimination of deformation temperatures is less effective due to the fact that, when heated, the form tends to its unstressed state.

At the time of publication of this patent, the separation accuracy in units of temperature was about ±3.5 °C and in units of extensions ±35 με at the end of a fiber sensor line 8.55 m long.

Many years have passed since the publication of this patent: detector sensitivities have increased, laser frequency tuning accuracy has increased, and new signal processing methods have appeared, including those using artificial intelligence. All of this will be discussed in the section below.

Thus, in [84], SMFs with standard and reduced cladding diameter were used. The temperature measurement accuracy of 0.3 K and the deformation of 8 µε were achieved with a line length of 50 m and a spatial resolution of 18 cm. This was possible due to the fact that these two types of fiber have different responses to temperature and deformation changes. In [85], the authors used a wavelength-division multiplexing scheme instead of a 50/50 splitter. The temperature measurement accuracy of 1.4 K and the deformation of 20 µε were achieved with a spatial resolution of 10 cm.

Another approach, as in the patent above, is to use PMF instead of SMF [86]. Then, the second quantity (in addition to the shift of the RBS found from the cross-correlation with the spectrum of the undisturbed state) becomes the spread of the RBS corresponding to the propagation of radiation along the fast and slow axes of the fiber. The latter, as in the pioneering works, can easily be obtained by finding the maximum of the autocorrelation function (either of the two; the autocorrelation function has two maxima, one of which corresponds to the shift of the spectrum of the fast axis to the spectrum of the slow axis, and vice versa for the other). The two experimentally determined quantities Δν_1_ and Δν_2_ are converted into temperature and strain using a relationship similar to Equation (23). In this work, the accuracy of temperature measurement of 0.1 K and strain of 1 µε were achieved with a line length of 180 m and a spatial resolution of 2.5 mm. It is obvious that the authors used an approach quite close to what was described in the patent above, but their temperature and deformation separation characteristics were much more accurate. This may be due to the fact that sometimes old proven methods, implemented on modern setups and components, show more impressive results.

In [87], the second measured disturbance was not strain but humidity (no mechanical stress was applied, but it was created via the polyimide sheath when moisture was collected), and a measurement accuracy of 0.5 K and 3% humidity was achieved. The fiber sensor was an HiBi-PM with a polyimide coating. Either the strain caused by humidity changes applied to the fiber or the temperature changes contribute to the different spectral responses in the cross-correlation and autocorrelation results of the Rayleigh backscatter signals.

**Figure 15:**
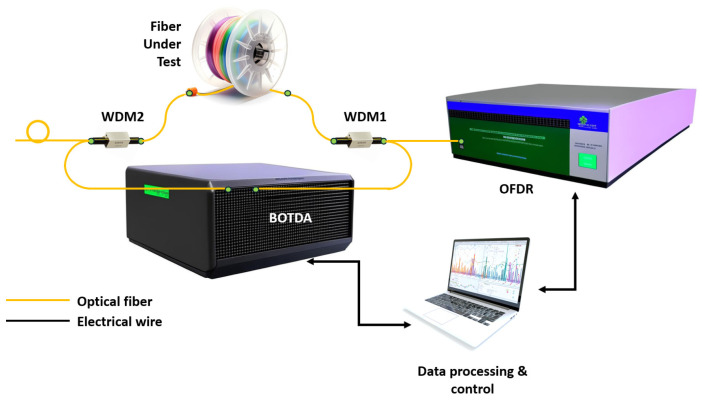
Schematic diagram of the setup proposed in [88]. WDM: wavelength division multiplexer.

Another approach is to simultaneously measure the shift of the RBS Δν_1_ and the shift of the Brillouin scattering spectrum Δν_2_ [88]. The scheme is shown in Figure 15.

The two measurement subsystems are connected by a 1310/1550 nm wavelength division multiplexer (WDM1). WDM2 is used to extract the TLS beam from the fiber loop after it passes through the FUT; otherwise, the RBS could be distorted by reflections on the Brillouin optical time-domain analyzer (BOTDA) elements such as the attenuator and isolator. A small loop is made at the end of the 1550 nm port of WDM2 to reduce the Fresnel reflection. The BOTDA subsystem is started first, and then, immediately after the BOTDA sources are put into standby mode, the OFDR subsystem is started. Δν_1_ and Δν_2_ are converted to temperature and strain using a relationship similar to Equation (23). The authors achieved a temperature measurement accuracy of 1.2 K and a strain of 15 µε with a line length of 92 m and a spatial resolution of 50 cm.

**Figure 16:**
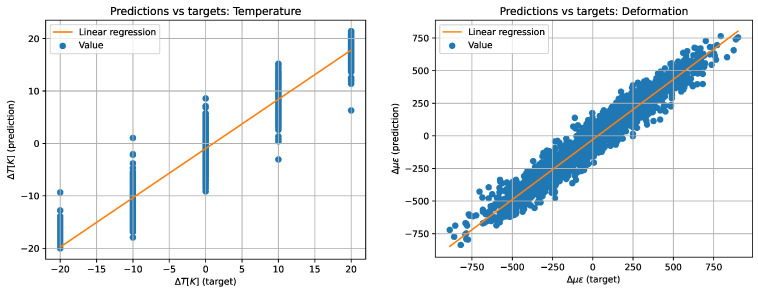
Two-dimensional representation of predicted and target values and linear regression for temperature (**left**) and strain (**right**) data.

Finally, in [89], an approach is used that is not based on relation (23). Instead, a phase-polarization analysis is used. In some ways, this is similar to the use of a PM fiber, but now the fast and slow axes are not created by the fiber structure; they are the result of the application of stresses. Since it is not possible to construct an analytical relationship between the output values of the photodetectors and the applied impacts, machine learning was used. At the training stage, the fiber was exposed to four different temperatures and five deformations. The use of the system made it possible to effectively obtain 2000 “independent” signals from different points of the fiber in each experiment. The work demonstrates the fundamental possibility of separating temperature and deformation, while the error in measuring temperature was less than 2 K, and the deformation was less than 100 µε. This is shown graphically in Figure 16.

It should be noted that the authors of this work make an important step in the transition from an artificial neural network as a black box to the so-called explainable artificial intelligence. This approach allows making the decision via the neural network understandable to humans. Using a special approach, the algorithm allows visualizing the weighting coefficients that characterize the features that the artificial intelligence is guided by. In the future, this will help to build various hypotheses and, based on them, obtain some analytical dependencies. Nevertheless, this approach can allow one to trace the “train of thought” of the neural network, but at this stage of the study, it is not possible to get a clear analytical dependence of the initial data and separated temperatures and deformations.

A great future can be seen in the combined use of analytical, including correlation algorithms, and methods of explainable artificial intelligence and image processing methods. It is also encouraging that methods for qualitative separation of the influence of temperatures and deformations on a distributed fiber sensor are also being created in systems based on OTDR. Perhaps the combination of OFDR and OTDR approaches, which will be discussed in the next section, will provide new research and engineering information—including that related to distinguishing thermal and deformation effects.

## 7. Convergence of OFDR and OTDR Technologies

OFDR technology differs from OTDR in that it allows absolute static measurements, including determination of the reflectors positions and physical quantities affecting the fiber. At the same time, OTDR has advantages in operating over long distances and high speeds. To combine the advantages of both techniques, instruments that combine pulsed sensing with wavelength tuning within one pulse or between them were created.

The main purpose of introducing such setups is their sensing length, comparable to classical OTDR systems capable of operating at distances of several tens of kilometers. An additional advantage is reduced requirements for the coherence length of the laser source, since it is sufficient to exceed it above the length of the pulse propagating along the fiber, thereby reducing the influence of laser phase noise. However, it should be noted that when measuring over long distances, the spatial resolution decreases due to an increase in the amount of data to be digitized or due to a drop in radiation energy as the pulse duration decreases.

Methods that use a pulsed setup with a wavelength-tunable radiation source are divided into systems that use chirping within the pulse—the method of time-gated digital OFDR (TGD-OFDR), and scanning utilizing pulses with different wavelengths—scanning OTDR.

### 7.1. TGD-OFDR Technique

**Figure 17:**
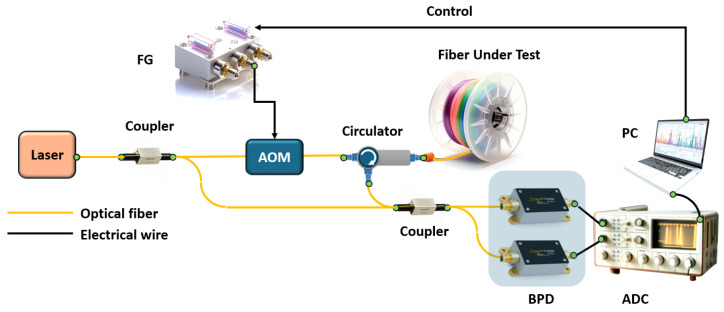
Schematic diagram of the TGD-OFDR system. FG: function generator; AOM: acousto-optic modulator; BPD: balanced photodetector; ADC: analog-to-digital converter; PC: personal computer [90].

Thus, a setup based on a new principle—TGD-OFDR—was presented in 2015 [90]. Its schematics can be seen in Figure 17.

The following numerical parameters of the equipment were presented in the work. A fiber laser (NKT, E15 – NKT Photonics A/S, Birkeroed, Denmark) with a nominal linewidth of 1 kHz was used. The AOM was driven via a function generator (Tektronix AFG3252C – Tektronix, Inc., Beaverton, Oregon, USA) with a frequency range of 150 MHz to 250 MHz within an 8 μs time window. To increase the frequency range, a modulator double-pass circuit was used, which provided tuning from 300 MHz to 500 MHz, with a frequency sweeping speed of up to 25 THz/s. The full width at half maximum (FWHM) of the pulse spectrum was 84 MHz, which corresponded to a theoretical spatial resolution of 1.2 m. The high-speed ADC (NI, 5185 – National Instruments Corporation, Austin, Texas, USA) had a sampling rate of 3.125 GS/s and 8-bit resolution. A pair of high-speed balanced photodetectors (Thorlabs, PDB480C – Thorlabs, Inc., Newton, New Jersey, USA) was used to receive beat signals from two orthogonal polarization states. The FUT consisted of four spools of single-mode fiber with a total length of 110.7 km. It can be seen that in general the optical configuration is similar to the OFDR system, with the exception that the laser wavelength is tunable within a pulse duration of 8 microseconds.

**Figure 18:**
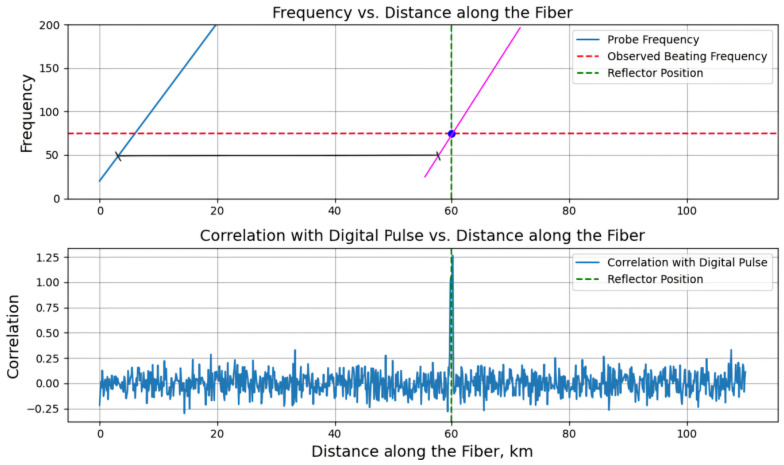
Frequencies of the beat signals received via a balanced photodetector (BPD) (**upper picture**), and the two equivalent references (**lower picture**) [90]. Pink line designates the digitally synthesized shifted probe signal.

The signal processing algorithm is similar to classic OFDR, which includes the use of an FFT, but with the FUT splitting into virtual segments corresponding to the pulse length. Although beat signals arising from reflections at adjacent points may overlap in time, their instantaneous frequencies are always different. The temporal position of the pulse and its corresponding scan frequency create a time-frequency display from which the position of each reflector can be determined. After receiving the trace, a correlation is performed with a digital pulse, which reconstructs the parameters of the radiation launched into the fiber, including its duration and wavelength tuning. The maximum value of the correlation function is observed when the time positions of both signals coincide, which corresponds to the location of the reflector in the fiber, as illustrated in Figure 18.

The figure demonstrates the beatings generated by reflections at different locations along the FUT having the same modulation frequency but occurring in different time windows depending on the time delay.

The reference signal *S_ref_* = cos(*πγt*^2^), where *γ* is the laser frequency tuning rate, is a digital copy of the pulse launched into the fiber and is used for comparison with the received signal. This standard matches the modulation frequency of the signal under test, which provides accurate analysis of time delays and frequency shifts of reflected signals, allowing accurate mapping of distances to reflections in the fiber. The difference in frequencies between the detected beating and the reference signal is directly proportional to the corresponding time delay *τ_d_* and frequency *F* = *γτ_d_*.

Thanks to the ability to work in a short pulse time window, it becomes possible to carry out fast scanning at speeds of up to 25 THz/s, limited only by the sampling frequency of the ADC. The high-frequency tuning rate also allows for effective suppression of phase noise from the laser source, as it reduces the laser coherence time requirement and reduces the influence of external disturbances due to shorter interaction times during pulse propagation. However, frequency tuning requires the use of high-speed signal generators and ADCs with high sampling rates. Moreover, it generates large amounts of data that must be efficiently processed and stored.

Another research group [91] used a 90-degree optical hybrid detector to extract phase information when studying the effect of vibration on the sensor. The method provided a minimum measurable vibration acceleration of 0.08 g. Since an ADC with a sampling frequency of 416 MHz and a resolution of 10 bits was used, it was possible to achieve a spatial resolution of 3.5 m with a maximum measured vibration frequency of up to 600 Hz and a measured fiber length of 40 km.

In addition to AOM-based scanning setups, TGD-OFDR setups can also use a current-controlled distributed feedback (DFB) laser. For instance, an approach to achieving high spatial resolution in the TGD-OFDR systems is presented in [92]. The proposed setup used an internally modulated DFB laser as a frequency-modulated light source that can reach a wide frequency modulation range up to 1.1 GHz. To compensate for the DFB laser frequency scanning nonlinearity, a pre-distorting system based on a Mach–Zehnder interferometer and Hilbert transform is used, which is described below. The pre-distorting system operates as a closed-loop feedback system and analyzes the frequency scanning characteristics of the DFB laser for nonlinearity compensation. Experimental results obtained using such a laser have demonstrated a spatial resolution of 18 cm on a 74 km FUT. As the authors claim, it was the best reported resolution for an OFDR system with this FUT length at that time.

Besides the basic signal processing algorithm in TGD-OFDR methods, the frequency of the probe pulse may be decomposed into overlapping frequency bands, to suppress phase noise and fading problems. The bands are processed independently of each other and collected together after digital decoding [93]. Thus, the frequency domain is divided into three overlapping bands for each of two polarization components (p and s). In this case, the data were collected over an interval of 20 cm in length, and the spatial resolution—the minimum distance at which the system can distinguish two individual events—was 1.8 m. This method also allows one to change the spatial resolution via post-processing, which makes it possible to vary it from 1.8 m to 13.5 m. For example, when monitoring submarine cables, the method made it possible to measure signals at a distance of up to 80 km with a spatial resolution of 1.8 m. The background noise level makes it possible to detect sound waves with an amplitude of 12.6 pε and higher.

### 7.2. Scanning Coherent OTDR (COTDR) Technique

**Figure 19:**
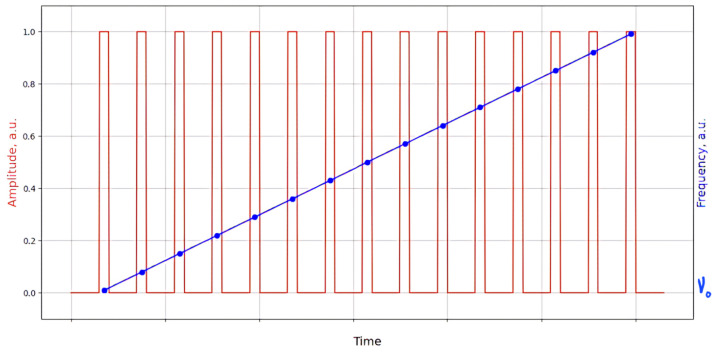
Schematic of the pulse generation and frequency sweep approach [94].

Another method of combining the COTDR and COFDR ideas is to tune the laser frequency from pulse to pulse. The setup operates like a classic phase-sensitive OTDR, in which the probe pulses have different wavelengths (Figure 19).

Optical pulse frequencies are defined as *ν_p_* = *ν*_0_ + Δ*ν*_p_ with discrete frequency steps Δ*ν*, where Δ*ν_p_* = *p*Δ*ν*, and *p* = 0, 1, 2… *m*. The backscatter traces for *m* + 1 values of the optical pulse frequency are measured during one laser scanning period *τ_s_*.

The method is based on the fact that the impact on the optical fiber, expressed as a change in the optical path between Rayleigh scatterers, can be compensated for by changing the wavelength of the laser source. This is possible because the interference equation uses a dimensionless phase quantity, where the optical path difference between the scatterers is normalized to the wavelength. Interferometric backscattering power:

(27) $$I_{coh}(t,t_{z})=\sum_{i=1}^{N}\sum_{j=1}^{N}r_{i}r_{j}\cos\left(\frac{2\pi }{\lambda }\Delta L_{i,j}(t)\right)$$

where *I_coh_*(*t*,*t_z_*) is the coherent component of the scattering power, *r_i_* and *r_j_* are the reflection coefficients of the scatterers, *λ* is the wavelength of the optical signal, and Δ*L_i_*_,*j*_(*t*) is the optical path difference between the scatterers *i* and *j*.

Since there is no AUX and the signal at each time moment is provided by the mutual interference of reflectors within a width of a light pulse passing through the fiber, classical algorithms for determining the position of reflectors based on the beat frequency are not suitable. However, it is possible to apply a correlation algorithm similar to that used in cross-correlation OFDR signal processing. As a result, to make absolute measurements, it is necessary to compare the unperturbed reflection with the reflection under the influence of a measured physical parameter, such as strain or temperature, which requires the use of a widely tunable radiation source to record the measured parameter over wide ranges.

A general disadvantage of this method is that the spatial resolution is limited by the duration of the light pulse, which is several meters along the fiber and cannot be significantly improved due to the consequent reduction in the total energy introduced into the fiber. Also, compared to OFDR, the disadvantage is the increased measurement time, since, in addition to waiting for the full frequency tuning cycle to complete at each frequency, it is necessary to wait for the signal to travel back and forth through the FUT, which is a significant amount for lengths of several tens of kilometers.

As in the case of OFDR, there are methods in which cross-correlation is carried out in the time domain and in the frequency domain, as well as a combination of both. In the first case, data for correlation are collected over time, which is equivalent to collecting data over distance along the fiber. However, to achieve the clear correlation function and to reduce the probability of inaccurate measurements, a sufficiently large number of measurement points is required, which, with a limited signal digitization speed, can lead to a deterioration in distance resolution. Therefore, it is preferable to use the second method, frequency correlation data collecting, since acquiring a large amount of frequency data is much easier, although it may reduce system performance due to the need to scan multiple laser frequencies.

**Figure 20:**
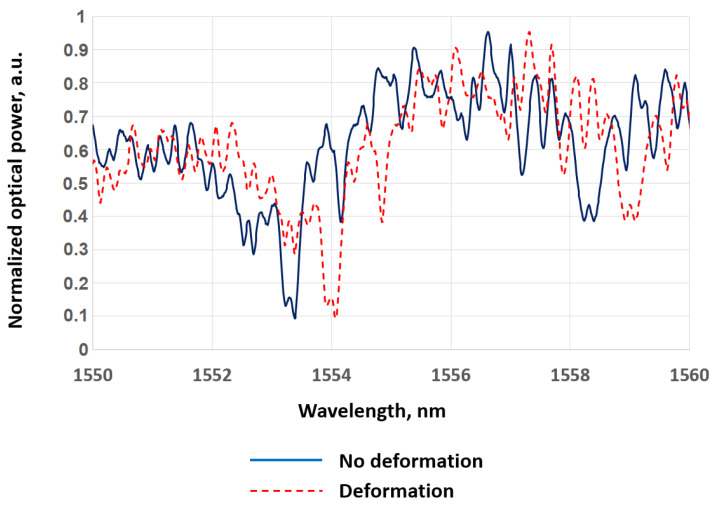
RBS for initial conditions at a specific fiber location for the case of with (red dashed line) and without (blue line) FUT deformation. Adapted from [95].

Typical spectra for the still reference state and the impacted one are shown sweeping across the laser frequency in Figure 20.

The spectral shift is determined via cross-correlation or the least squares algorithm to accurately determine the magnitude of the effect. Comparison of two spectra can be carried out both for spectra obtained at successive moments of time and via comparison with the unperturbed (reference) state of the fiber. In the latter case, measurements are performed in absolute values but require a wide wavelength tuning range corresponding to the full range of the measured parameter.

A set of wavelengths in a scanning OTDR setup, varying the frequency of each pulse, is usually generated using the same hardware methods used in OFDR. To change the wavelength in OFDR, programmable lasers, modulation using an external modulator, and systems with an external cavity diode laser can be utilized. However, since the scanning OTDR setup, unlike OFDR, does not require continuous scanning, but only changes the frequency step by step, sometimes interesting methods are used to specify a series of pulses of different frequencies. Thus, a new approach for obtaining pulses with a frequency shift of each of them, based on the use of frequency shift loops (FSL), is proposed [96]. The FSL structure is a frequency-shifted fiber optic loop, creating multiple delayed and frequency-shifted versions of the local oscillator. This method offers advantages in terms of system stability, measurement accuracy, and ease of implementation compared to other frequency scanning methods.

Since the signal processing algorithms are similar to the cross-correlation methods used in OFDR, the details of which have already been described above, in this section we will not go into technical details but will concentrate on some interesting aspects and experimental results. For example, second-order subpixel interpolation can be used to improve the accuracy of strain measurement [94]. The results are as follows: the linearity of the strain response was confirmed over a wide range of amplitudes from 47.5 pε to 778 nε, which is significantly lower than the equivalent frequency quantum value Δε = 79 nε at a fiber length of 970 m. The study used a DFB laser diode with a spectral width of 1.3 MHz, a central wavelength of 1549.89 nm (193.36 THz), and a tuning range of 4.15 GHz.

In addition to laser radiation sources, it is also possible to use tunable filters (so-called low-coherence systems) to scan a wide range of wavelengths. Thus, a broadband pulsed laser diode Superlum SLD 761 (Superlum Diodes Ltd., Carrigtwohill, Ireland) with a spectral width of 40 nm (FWHM) and a narrow-band tunable MEMS filter DiCon Fiberoptics MTF500B (DiCon Fiberoptics, Inc., Richmond, CA, USA) with 0.17 nm spectral width (FWHM) showed relatively good results [95]. The contrast of the interference pattern was approximately 0.45. A General Photonics PSM-002 (General Photonics Corporation, Chino, CA, USA) polarization scrambler was also used to eliminate polarization effects that could destabilize data acquisition. The signal from the photodetector was digitized with an 8-bit ADC with a sampling rate of up to 400 MHz. The spectral cross-correlation method allowed the authors to achieve the following parameters: root mean square noise was 0.3 °C for a 25 km fiber. The dynamic range of measurements reached 2000 µε. The noise level (RMS error) was 2 µε. The spatial resolution was 1 m with a data collection time of 10 min. The pulse duration was 6.5 ns. Discussing the scanning reflectometer with a low-coherence radiation source, one should consider the article [97]. It is reported that, through the use of Raman amplification and remotely pumped EDFAs, it is possible to achieve a measurement distance of up to 85 km. The strain measurement range reached up to 500 µε, which corresponds to 56 °C in temperature units. The uncertainty in the deformation measurements was 3.8 µε, and for the temperature measurement, it was 0.42 °C, with a spatial resolution of 2.6 m.

As with COFDR, scanning COTDR can use phase information to determine the shift of the spectra. Thus, a phase cross-correlation (PCC) method that uses spectral phase information is proposed to reduce significant errors in frequency-scanned phase-sensitive optical reflectometers [98]. This method is based on analyzing the instantaneous phase of spectral data instead of amplitude values. In the traditional cross-correlation technique, spectral samples with high intensity are weighted more heavily, which can lead to high false correlation peaks and hence large errors. The PCC method solves these problems by using the phase information of the spectra. Obtaining the phase of the signal is possible using hardware, for example, using a 3 × 3 coupler or an I/Q detector. In the mentioned study, spectrum analysis was performed on a single receiver using analytical signal construction. Since this method is often used in OFDR to extract the phase component of the signal in the measuring path, as well as for the AUX channel, it will be described more thoroughly.

For a real signal *s*′(*ν_i_*), the analytical signal *S*(*ν_i_*) can be represented in the following form:

(28) $$S(\nu_{i})=s^{\prime }(\nu_{i})+jH\left[s^{\prime }(\nu_{i})\right]$$

where *j* is the imaginary unit; *H*[*s*′(*ν_i_*)] is the Hilbert transform of the signal *s*′(*ν_i_*). Amplitude *a*(*ν_i_*) and phase *φ*(*ν_i_*) of the analytical signal can be expressed through the Hilbert transform:

(29) $$a(\nu_{i})=\sqrt{{s^{\prime }}^{2}(\nu_{i})+H{\left[s^{\prime }(\nu_{i})\right]}^{2}}$$

(30) $$\varphi (\nu_{i})=\mathrm{arctg}\left(\frac{H\left[s^{\prime }(\nu_{i})\right]}{s^{\prime }(\nu_{i})}\right)$$

And the phase cross-correlation function (PCCF) is determined through the measured and reference spectra *s_k_*(*ν_i_*) and *s*_0*k*_(*ν_i_*) as follows:

(31) $${\mathrm{PCCF}}_{s_{k},s_{0k}}\left(\delta \nu \right)=\frac{1}{2F}\overset{F}{\underset{i=1}{\sum }}{\left|e^{j\phi_{k}\left(\nu_{i}+\delta \nu \right)}+e^{j\phi_{0k}\left(\nu_{i}\right)}\right|}^{\chi }-{\left|e^{j\phi_{k}\left(\nu_{i}+\delta \nu \right)}-e^{j\phi_{0k}\left(\nu_{i}\right)}\right|}^{\chi }$$

where *ϕ_k_*(*ν_i_*) and *ϕ*_0*k*_(*ν_i_*) represent the instantaneous phases of the measured spectrum and the reference spectrum, respectively; *δν* is the frequency shift; *F* is the number of scanned frequencies; *χ* is the PCCF power indicator, which controls the sharpness of the transition from correlated to uncorrelated values. When *χ* = 2 the equation simplifies as follows:

(32) $${\mathrm{PCCF}}_{s_{k},s_{0k}}\left(\delta \nu \right)=Re\left\{\frac{1}{F}\overset{F}{\underset{i=1}{\sum }}\left|e^{j\phi_{k}\left(\nu_{i}+\delta \nu \right)}+e^{j\phi_{0k}\left(\nu_{i}\right)}\right|\right\}$$

This simplified form can be implemented efficiently using FFT, where *Re*{·} denotes the real unit operator. This function is normalized, which avoids the influence of high-intensity spectral components and ensures that all spectral points are evenly weighted. The experiments were carried out using 5.63 km FUT. A spatial resolution of 1 m was obtained. For temperature variation, the use of the traditional cross-correlation method resulted in 59 large errors; the use of the phase cross-correlation method resulted in frequency shifts correctly calculated along the entire FUT.

Also, in addition to the cross-correlation method, the least squares method is used when comparing spectra. So, a scanning OTDR with the following parameters is reported [99]. The spatial resolution was 20 cm, achieved through the use of short 2 ns optical pulses. The sampling rate was 27.8 kHz with a fiber length of 55 m. The maximum measurable strain range was 80 με with a noise level of less than 1.8 nε/√Hz for vibrations below 700 Hz and less than 0.7 nε/√Hz for higher frequencies. The number of frequency scanning steps was 60.

A review of the literature on this topic has shown that methods combining OTDR and OFDR approaches are promising for measuring long fiber lengths.

## 8. System Simplification and Cost Reduction

One of the main problems in OFDR technology is associated with the need to use TLSs, which must satisfy the following criteria:

1. Long coherence length;
2. High degree of optical frequency sweeping linearity;
3. Wide spectral range.

A long coherence length is necessary to observe interference. At a minimum, this means that the radiation at each moment of time must be of a single frequency. Sweeping linearity is required for further signal processing—performing the FFT. With a nonlinear dependence of frequency on time, the time samples of the measurement will correspond to non-equidistant frequency samples, which, in turn, will lead to a broadening of the events on the OFDR-trace. A wide range, as noted above, is necessary to obtain high spatial sampling of the traces. Laser sources that meet the above criteria are expensive, which ultimately limits the widespread use of OFDR technology. For this reason, approaches are being developed that overcome the limitations associated with expensive TLSs.

Currently, a large number of methods for tuning the optical frequency of a laser are known. Typically, they are all based on tunable filters such as diffraction gratings [100], FBGs [101], various interferometers [102], etc. However, in most cases, such filters do not allow obtaining single-frequency lasing, because the spectral width of the tunable filters is quite large. To obtain lasing on one longitudinal mode of the resonator, it is necessary to reduce the length of the resonator (to separate the resonator modes in the frequency domain) and/or to reduce the spectral width of the selector used. For example, these two conditions are simultaneously satisfied in widely available DFB lasers, which have proven themselves in telecommunications applications. To adjust the laser frequency in such lasers, current modulation is used. However, in this case, when the frequency is adjusted, frequency hops may occur, and its dependence may be nonlinear. In [103], the issue of the uncontrolled frequency hops’ influence at the level of 0.1 nm in an OFDR system based on a semiconductor DFB laser with current tunability is considered. In the simulation, the sweeping with a range of 1 nm was assumed to be linear. Using simulation, the authors show that even in the case of frequency hops, such an OFDR system allows one to determine the coordinates of reflectors on the trace with high accuracy. Considering the problem of strain measurement, frequency hopping introduces an error at the level of 1% of the applied strain. The presented setup with a DFB laser was experimentally verified in [104] during the deformation measurement. Standard single-mode fiber and fiber with induced Rayleigh scatterers were used as sensitive elements. The BEOF fiber improves the SNR of the traces by more than 50 dB. Although strain response was observed even for conventional fiber, the best results were obtained for fiber with induced Rayleigh scatterers. The deformation measurement error was 1.5 με with an impact of 50 με at a spatial resolution of 24 mm over a fiber length of 1 m.

In the two abovementioned works, the DFB laser frequency sweeping nonlinearity correction was applied to already measured signals, taking into account data from the AUX. In another work [105], in an OFDR system based on a semiconductor DFB laser with current tunability, it is proposed to correct the nonlinearity by adjusting the control current. The laser radiation enters the AUX. To do this, the digitized laser signal, after passing through an AUX, is fed to an FPGA-based processing module. The module generates a control signal that is sent to the DFB laser, thereby closing the feedback loop. It was found that the sweeping nonlinearity is 16.55% and 0.078% for the case with open and closed feedback loops, respectively. The proposed system was tested for the case of the reflectors’ position measurement in a 45 m long FUT. In the case of nonlinearity correction, the position measurement error was about 4 mm. Without adjustment, the measurement error was 22 cm for the shorter 15 m FUT.

**Figure 21:**
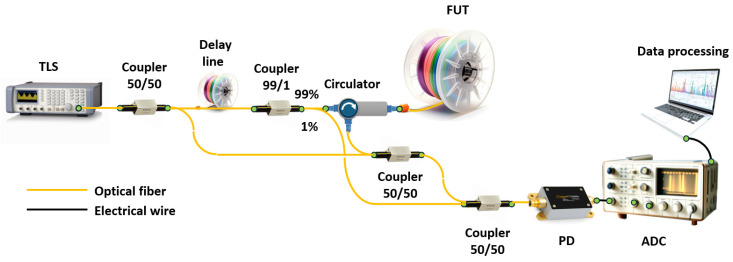
OFDR with a combined main and AUX interferometer. Adapted from [106].

The use of an AUX to correct the nonlinearity of frequency tuning also requires an increase in computing resources. At the very least, this requires an additional photodetector and a corresponding ADC channel. In [106], it is proposed to use the same photodetector and ADC as for measuring the main signal coming from the FUT. To do this, a 3.6 m-long delay line is added to the setup between the AUX and main interferometer (Figure 21). This allows both signals to be measured using single-channel detection. The approach was experimentally tested using a 50 m-long FUT when measuring temperature and deformations in the fiber. Spatial and frequency sampling in this case were 45 mm and 2.3 GHz, respectively.

**Figure 22:**
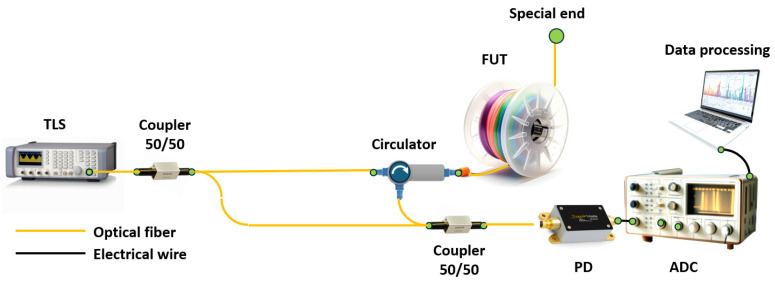
OFDR with reflector in the main interferometer. Adapted from [108].

A similar problem was solved in [107]. To accomplish this, the authors propose the use of reflection data from the end of the FUT. Using the known characteristics of the final reflector, the authors reconstruct the frequency-versus-time dependence, which is subsequently used to correct nonlinearity. In other words, the main interferometer with a point reflector at the end is also an AUX. Moreover, the setup proposes splitting the beat signal into two using an analog band-pass filter. The low-frequency and high-frequency parts of the signal are used to calculate the OFDR trace and the dependence of frequency on time. It is important to note that the high-frequency part did not require an additional digitization channel and was implemented using simpler circuitry. The disadvantage of this approach is that the maximum FUT length is limited, which is determined by the bandwidth of the filter used. Practically the same idea involving the use of the end-face reflector was proposed in [108]. To do this, the authors propose to use reflection data from a specially prepared end of the line in the form of a hemisphere (Figure 22). Unlike the previous case, the authors split the output digitally to get the complete signal. The setup was tested to measure temperature over a FUT length of up to 170 m with a spatial sampling of 5 mm.

When the absolute OFDR measurements are required, in addition to the AUX, a gas cell could be added to the setup as a wavelength reference [109]. This leads to the necessity of the use of one more measuring channel. In [110], the authors proposed combining the AUX channel and the data channel for the signal after passing through the gas cell. Despite combining the two channels into one channel, they managed to separate the data and use them for absolute measurements. In particular, this allows for increased data reproducibility in OFDR.

The setups discussed above were based on semiconductor lasers. Single-frequency tunable lasing can also be obtained in DFB fiber lasers. In this case, frequency tuning can be achieved using mechanical stretching of the laser cavity. A feature of such lasers is that when sweeping the laser frequency, frequency hops are excluded [111]. In [112], an erbium-doped fiber DFB laser with a bandwidth of 10 kHz was used to build the OFDR system. Lengthening the resonator by 0.09% via piezoceramics provided a tuning of ~1 nm. The authors show the possibility of the reflectors’ locations over a FUT length of 150 m with a spatial sampling of 16 cm. The relatively high spatial sampling was a consequence of the limited number of measured points, which also limited the sweeping range to ~0.01 nm. Good agreement between the calculated and experimental data indicates a high degree of the sweeping linearity (at least within 0.01 nm). For this reason, assisted measurements do not require the use of an AUX. It is difficult to expect a high quality of sweeping for large tuning ranges. In [113], stretching the DFB fiber laser using a magnetic field in the solenoid by 1.2% provided a wavelength tuning of 14 nm. In this case, the authors already needed an AUX to adjust the sweeping function by generating a control signal. In this case, the deviation from the linear tuning function was 0.14%. A similar approach to adjusting the tuning of a piezoceramic-based DFB fiber laser was demonstrated in [72]. The authors achieved good linearity of sweeping at high speed. Wavelength scanning in the range of 0.01 nm occurred in 25 ms. This allowed them to interrogate long FUTs (up to 200 km). and the measurements’ elapsed time was 5 ms. However, the spatial sampling was 72 cm due to the relatively small tuning range. For a line length of 20 km with a spatial sampling of 10 cm, the measurement took 25 ms.

**Figure 23:**
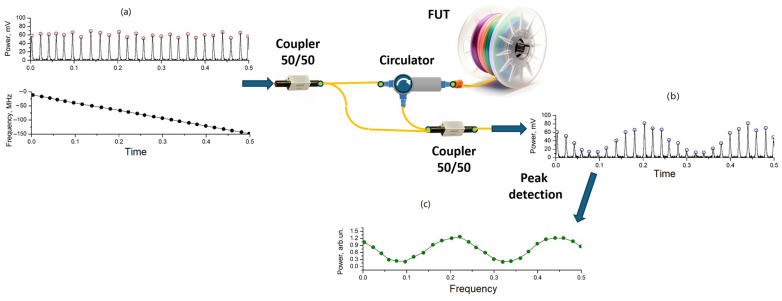
The operating principle of OFDR based on a self-scanning fiber laser. (**a**) Laser radiation, consisting of a sequence of pulses with a fixed discrete frequency entering the main interferometer; (**b**) output of the interferometer, the sequence of pulses acquires an amplitude envelope; and (**c**) the extracted envelope has equidistant frequency samples.

An alternative approach to obtaining single-frequency tunable lasing in fiber lasers instead of DFB structures is the use of dynamic population gratings [114]. A feature of dynamic gratings is their finite lifetime (about several ms). In contrast to classical fiber gratings, dynamic population gratings are formed only in active fibers (as a rule, doped with rare earth elements) under the influence of a standing wave of radiation. These gratings can have a length from several units to several tens of meters. Due to this, they have high spectral selective properties up to filtering one longitudinal mode of the resonator. In this case, maximum reflection can be achieved for radiation with an optical frequency different from the frequency that recorded this grating. In this case, a self-induced sweeping of the laser frequency can be observed in a fiber laser without any control elements. In this case, the authors talk about so-called self-scanning lasers (see references in [114]). The ability to achieve large tuning ranges (up to 24 nm) and high coherence (bandwidth up to 1 MHz) in self-scanning lasers opens up new prospects for low-cost OFDR systems. Another important feature of such lasers is that scanning occurs only along the modes of the laser cavity, which eliminates the need to use an AUX. In other words, the AUX in such a laser is the laser interferometer itself. As a result, the primary signal in such a system is the dependence of the radiation amplitude on the serial number of the resonator mode (Figure 23). This signal format is best suited for FFT calculations without additional resampling. It is worth noting that, unlike classical OFDR, where the reference and scattered waves have a difference in frequencies during interference, in this case, the frequencies for the waves will be the same. However, a phase difference appears between the waves, the magnitude of which depends on the wavelength of the radiation. The first demonstration of an OFDR system based on a self-scanning laser was presented in [115]. The work demonstrated the possibility of measuring the position of point reflectors along a FUT of up to 9 m. Later, in [116], using the fiber with an array of weakly reflective FBGs with the same resonance, the possibility of measuring temperature was shown. Optimization of the main interferometer in [117] made it possible to measure weak signals at the level of Rayleigh scattering in a conventional fiber. In this work, the authors obtained an OFDR system sensitivity of ~−120 dB/mm. The authors were able to measure the temperature in a 9 m long FUT with a spatial sampling of 40 mm.

Further improvements in OFDR-like systems’ SNR can be achieved using quasi-continuous self-scanning lasers, for which it becomes possible to accumulate data for each individual frequency [118]. This approach makes it possible to increase the SNR by up to 20 dB compared to previous OFDR schemes based on self-scanning lasers. The possibility of measuring distances with high accuracy using a self-scanning laser without using a main interferometer is shown in [119,120]. In this case, the FUT was part of the laser cavity. A change in its length manifested itself in a change in the frequency of intermode beats. However, this approach allows one to determine the distance only for one reflector, and not their longitudinal distribution.

To conclude, it should be noted that the main ways for the OFDR systems’ cost reduction are using a common semiconductor and fiber DFB lasers and self-scanning fiber lasers, as well as the reduction in measurement channels. Despite these simplifications, OFDRs are still able to produce highly technical results that can be used in various applications.

## 9. Discussion and Conclusions

In this review, both the most important scientific and technical achievements in the field of creation and development of OFDRs over the past few years, as well as some pioneering works from the past years, were mentioned. This narration is provided with the main fundamental equations and theoretical principles. Beyond the scope of this review are numerous applications of systems based on the OFDR method—such works are published regularly, and individual studies related to one or another engineering problem are published almost daily. This is a good characterization of the scientific and technical communities’ demand for technologies of this kind. Industrial enterprises around the world also demonstrate strong demand for commercial OFDRs. This fact explains the availability of stable funding for applied research in the development of new OFDR designs, special fiber sensors, new RBS processing algorithms, and the creation of special neural networks for extracting temperatures and strains.

It should be noted that the sensing component of the OFDR prevails over the metrological one. In fact, the last significant works on this topic appear to be the publications of the researchers from Luna Innovations, which published their first work on fiber and photonic circuit monitoring back in the early 2000s. From this, it can be withdrawn that OFDR, as a tool for studying the internal structure of various fiber circuits, has ceased to change significantly over the past 15–20 years. Recent publications are only about the study of the fiber-optic gyroscopes’ coils, where it is necessary to increase the sensor interrogation range due to the length of the optical fiber, as well as about the study of birefringence and other properties of special fibers [121]. However, signal processing techniques are constantly being refined to improve the spatial resolution of OFDR. This is very important for metrology of photonic and integrated optical circuits, which have small dimensions and complex topologies, as well as for sensor applications, where the resolution of the OFDR trace will ultimately determine the spatial sampling of the distributed sensor.

The much greater popularity of sensor applications can naturally be explained by the prevalence of engineering problems solved using sensor OFDRs.

Among the works on OFDR characteristic improvements, four large groups can be distinguished:

1. This group of works includes studies aimed at imparting special properties to the sensitive element. Thus, fibers with a changed aperture and mode field diameter or with a different composition of doping components, low-mode fibers, and multi-core fibers are used. As already noted, this is a rather expensive solution, and it is not applicable if an SMF-based fiber line is already installed for the object under study. In addition, the new opportunities offered by specialty fibers usually lead to undesirable consequences, such as reduced interrogation length. Moreover, the use of sensors, for example, to estimate the shape of an object using multi-core fibers, entails modification of the software, since processing data from multiple cores is actually a new engineering or even scientific problem;
2. The second group of works includes software modifications. Signal processing techniques have a significant impact on the accuracy of an OFDR. The most important of them are the laser frequency tuning nonlinearity compensation and temperature and strain extraction algorithms. Often, such studies are carried out on standard, commercial OFDRs, or using the author’s own laboratory equipment, which are structurally no different from well-known setups;
3. The optical setup is subject to modification. Here we can highlight two main tasks that OFDR hardware modifications are aimed at:
  3.1. Improving the performance of the instrument. This may include polarization channel separation, additional backscattered power amplification, I/Q detection, etc. The integration of OTDR and OFDR methods into each other to obtain improved performance and eliminate the shortcomings of both methods is also included in this category;
  3.2. Simplification of the instrument setup, which leads to a reduction in the market price of a commercial OFDR while maintaining the operational parameters required for various tasks. Since the most expensive element is a highly coherent TLS, more and more research is emerging on replacing this device with an element operating on new physical principles, for example, on the principle of frequency self-scanning;
4. A group of works in which the modifications mentioned in two or more groups above are made.

Based on the reviewed studies, a table can be compiled that contains the best achieved performance of individual OFDRs (Table 1).

Of course, it is worth noting that the parameters described in the table above cannot be achieved within a single system. However, in the last row of the table a kind of “winner” has been chosen that has a set of characteristics worthy of attention. It may sound surprising, but these impressive performances were obtained using a standard OFDR configuration. The system included a TLS, main, and AUXs. There was no gas cell or corresponding data recording channel in this system. The key to achieving these characteristics was the use of an artificial neural network, which was trained on simulated and hybrid data, taking into account all possible scenarios of impact on the sensor in the range from −10,000 to 10,000 με.

As already noted, the OFDR industry is largely driven by the demand for sensor tools [122,123,124]. In its turn, it is highly dependent on the development of technologies related to machine learning and artificial intelligence [125,126]. Neural networks quite easily process spectra and correlation functions that have a low SNR, helping to restore the signal where conservative methods seemed to be useless. Particular prospects emerge for the use of artificial intelligence methods in conjunction with image processing algorithms. RBS, presented in two-dimensional form, contain a lot of seemingly redundant information. However, practice has shown that very often information about neighboring points, and sometimes even points remote from the location being studied, makes a key contribution to the selection of weighting coefficients in the neural network algorithm, which can significantly expand the range of recorded values, increase accuracy, and add new possibilities for separating the impact of various factors on the sensor.

Combining OFDR and OTDR technologies, that is, the use of pulses with variable frequency, made it possible to maintain the metrological characteristics inherent in OFDR, although with some loss in spatial resolution with increasing range, which is typical for the OTDR-based methods. This approach represents a full-fledged alternative to the Brillouin reflectometry, while having greater sensitivity and operating speed due to the significantly larger reflected signal from Rayleigh reflectors compared to inelastic Brillouin scattering.

We believe that the current development of OFDR technology is just a starting point and is the beginning of a long journey that opens up truly impressive opportunities.

## Figures and Tables

**Table 1:** The best OFDR parameters achieved, according to this review.

Parameter	Units	Value	Comments	Ref.
Spatial sampling in back reflection measurements	μm	21.3	Obtained using the EFR method. Fiber length is 191 m with a 200 m optical path delay in the AUX.	[26]
Spatial sampling in sensing mode	mm	0.857	Obtained using a physics-driven neural network.	[66]
Lowest back reflection power registered	dB	−70	A high-sensitivity, highly integrated, and low-noise coherent receiver module based on germanium-silicon photonic chips was used.	[32]
Temperature measurement range	°C	Up to 800	Zirconia-doped fiber was used.	[49]
Strain measurement range	με	−10,000–10,000	Spectral segmentation method was used.	[63]
Accuracy of temperature measurement	°C	0.05	VNA-assisted OFDR was used; spatial resolution is 3 m.	[78]
Accuracy of strain measurement	με	0.25	The differential relative phase method is used in the OFDR.	[60]
Accuracy of temperature measurement when distinguishing temperature and strain	°C	0.1	A 180 m FUT and a spatial resolution of 2.5 mm; PMF fiber.	[86]
Accuracy of strain measurement when distinguishing temperature and strain	με	1		
Probing length in back reflection measurements	km	242	High-order optical phase-locked loop-assisted fiber laser with 4.3 cm spatial resolution; the backscatter measurement precision is 0.5 dB.	[35]
Probing length in temperature and strain measurements	km	5.63	A frequency-scanned φ-OTDR and phase cross-correlation were used.	[98]
Probing length in vibration measurements	km	64	Obtained using sinusoidal frequency sweep; detection of 400 Hz signal with a spatial resolution of less than 10 m.	[81]
Range of recorded vibration frequencies	kHz	Up to 50	Obtained by adding reference vibration sources to the FUT; 25 m FUT length.	[82]
Best set of parameters for a single system	Spatial resolution of 0.857 mm, impact detection accuracy of 0.91 με, and sensor length of 140 m.		Obtained using physics-driven neural network.	[66]

## Data Availability

Not applicable.

## Abbreviations

The following abbreviations are used in this manuscript:

ACF	Autocorrelation function
ADC	Analog-to-digital converter
AOM	Acousto-optic modulator
AUX	Auxiliary interferometer
BEOF	Backscatter enhanced optical fibers
BOTDA	Brillouin optical time-domain analyzer
BOTDR	Brillouin optical time-domain reflectometer
CCF	Cross-correlation function
COFDR	Coherent optical frequency-domain reflectomer
COTDR	Coherent optical time-domain reflectometer
DFB	Distributed feedback
DSB	Dual sideband
EDFA	Erbium-doped fiber amplifier
EFR	Equal frequency resampling
EOM	Electro-optical modulator
FBG	Fiber Bragg grating
FFT	Fast Fourier transform
FSL	Frequency shift loop
FUT	Fiber under test
FWHM	Full width at half maximum
MCVD	Modified chemical vapor deposition
OFDR	Optical frequency-domain reflectometer
OTDR	Optical time-domain reflectometer
PCC	Phase cross-correlation
PCCF	Phase cross-correlation function
PMF	Polarization maintaining fiber
PPNE	Periodic-phase-noise-estimated
RBS	Rayleigh backscattering spectra
RMS	Root mean square
SNR	Signal-to-noise ratio
SSB	Single sideband
SMF	Single-mode fiber
TGD-OFDR	Time-gated digital optical frequency-domain reflectometer
TLS	Tunable laser source
VNA	Vector network analyzer
WDM	Wavelength division multiplexer
φ-OTDR	Phase-sensitive optical time-domain reflectometer
